# Reversal of established liver fibrosis by IC-2-engineered mesenchymal stem cell sheets

**DOI:** 10.1038/s41598-019-43298-0

**Published:** 2019-05-02

**Authors:** Noriko Itaba, Yohei Kono, Kaori Watanabe, Tsuyoshi Yokobata, Hiroyuki Oka, Mitsuhiko Osaki, Hiroki Kakuta, Minoru Morimoto, Goshi Shiota

**Affiliations:** 1 Division of Molecular and Genetic Medicine, Graduate School of Medicine, Tottori University, 86 Nishi-cho, Yonago, Tottori, 683-8503 Japan; 2 KanonCure Inc., 86 Nishi-cho, Yonago, Tottori, 683-8503 Japan; 3 Research Initiative Center, Tottori University, 4-101 Koyama, Tottori, 680-8550 Japan; 4 Division of Pathological Biochemistry, Department of Biomedical Sciences, Faculty of Medicine, Tottori University, 86 Nishi-cho, Yonago, Tottori, 683-8503 Japan; 5 Division of Pharmaceutical Sciences, Okayama University Graduate School of Medicine, Dentistry and Pharmaceutical Sciences, 1-1-1, Tsushima-naka, Kita-ku, Okayama, 700-8530 Japan

**Keywords:** Regeneration, Liver fibrosis

## Abstract

Chronic hepatitis viral infection, alcoholic intoxication, and obesity cause liver fibrosis, which progresses to decompensated liver cirrhosis, a disease for which medical demands cannot be met. Since there are currently no approved anti-fibrotic therapies for established liver fibrosis, the development of novel modalities is required to improve patient prognosis. In this study, we clarified the anti-fibrotic effects of cell sheets produced from human bone marrow-derived mesenchymal stem cells (MSCs) incubated on a temperature-sensitive culture dish with the chemical compound IC-2. Orthotopic transplantation of IC-2-engineered MSC sheets (IC-2 sheets) remarkably reduced liver fibrosis induced by chronic CCl_4_ administration. Further, the marked production of fibrolytic enzymes such as matrix metalloproteinase (MMP)-1 and MMP-14, as well as thioredoxin, which suppresses hepatic stellate cell activation, was observed in IC-2 sheets. Moreover, the anti-fibrotic effect of IC-2 sheets was much better than that of MSC sheets. Finally, knockdown experiments revealed that MMP-14 was primarily responsible for the reduction of liver fibrosis. Here, we show that IC-2 sheets could be a promising therapeutic option for established liver fibrosis.

## Introduction

Chronic liver injury leads to liver fibrosis, which is the excessive accumulation of extracellular matrix (ECM). Moreover, advanced and established liver fibrosis results in liver cirrhosis. Especially, established liver fibrosis progresses to decompensated liver cirrhosis, for which medical needs remain unmet^1–6^. In addition, because liver fibrosis is strongly associated with the incidence of hepatocellular carcinoma (HCC)^7^, the development of anti-fibrotic therapies would not only cure liver fibrosis, but also suppress subsequent incidences of HCC.

Liver transplantation is the most effective treatment for liver cirrhosis^8^. However, it is not feasible for all patients because of donor scarcity. Stem cell therapy shows considerable potential as a treatment for liver disease^9,10^, and the therapeutic effects of mesenchymal stem cells (MSCs) have been studied in clinical research. Accordingly, MSCs are emerging as a promising cell source for the treatment of liver cirrhosis^11–15^. However, there is much room for improvement regarding the methodology of MSC transplantation to accomplish an effective therapeutic modality (e.g. optimal delivery route, sufficient number of MSCs, and extension of the survival of engrafted MSCs)^16^. Cell sheet engineering has attracted much attention to overcome these problems. This technology enables the transplantation of abundant cells without the risk of embolization or undesired cellular migration to other organs, as compared to the intravascular infusion of cell suspensions. Furthermore, transplanted cell sheets can be engrafted for a long term without losing their functions, as compared to that with the intravascular infusion of cell suspensions^17^. Indeed, in a myocardial infarction model, myoblast cells transplanted as a cell sheet suppressed fibrosis compared to that with cell infusion^18^. Therefore, tissue-engineered cell sheet transplantation should be an appropriate treatment for liver fibrosis.

Based on our observations that Wnt/β-catenin signaling is downregulated during the hepatic differentiation of MSCs and that suppression of this signaling axis causes the hepatic differentiation of MSCs^19,20^, we previously produced hepatic cell sheets from MSCs via treatment with hexachlorophene, a Wnt/β-catenin inhibitor, on thermoresponsive polymer-coated culture dishes^21^. Orthotopic transplantation of hexachlorophene-treated MSC sheets ameliorated carbon tetrachloride (CCl_4_)-induced acute liver injury^21^. In another previous report, we screened our synthetic chemical libraries to improve the performance of cell sheets, identifying IC-2, a derivative of the Wnt/β-catenin signaling inhibitor ICG-001. IC-2 proved to potently induce the differentiation of MSCs into hepatic lineages^22^. Recently, we reported that the therapeutic effect of IC-2-engineered cell sheets (i.e. IC-2 sheets) on acute liver injury is more potent than that of hexachlorophene-engineered MSC sheets^23^. In addition, it is not known whether IC-2 sheets have anti-fibrotic effects on liver fibrosis. In the present study, we clarified the anti-fibrotic effect of IC-2 sheets on liver fibrosis and its molecular mechanisms.

## Results

### Reversal of liver fibrosis via orthotopic transplantation of IC-2 sheets during chronic liver injury

**Figure 1:**
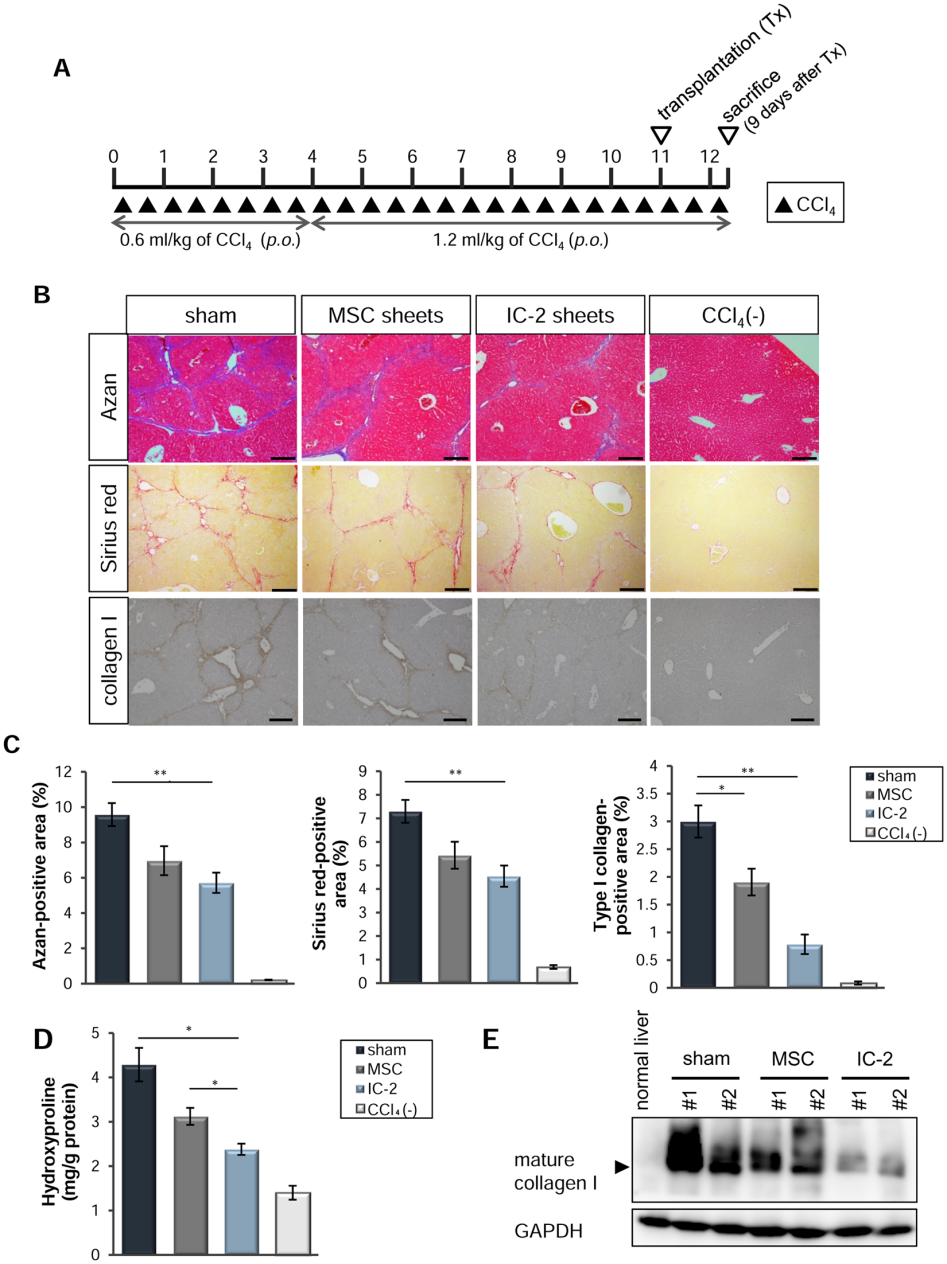
Suppression of hepatic fibrosis by IC-2 sheets on the ninth day after transplantation. (**A**) Protocol of the animal experiment. Abbreviations: *p.o*., *per os*; Tx, transplantation. “p.o.” indicates oral administration of CCl_4._ “Tx” indicates transplantation of cell sheets. (**B**) Micrographs of liver sections subjected to Azan staining (upper), Sirius red staining (middle), and immunohistochemistry for type I collagen (lower). MSC, mesenchymal stem cell. (**C**) The proportions of fibrotic areas among the groups (n = 7–9 except for n = 3 for CCl_4_(−) group). CCl_4_(−) indicates control mice without CCl_4_ intoxication. (**D**) Hydroxyproline contents in the liver (n = 6 except for n = 3 for CCl_4_(−) group). (**E**) Western blot of type I collagen in recipient liver tissues. The results are expressed as the mean ± S.E.M. Levels of significance: *P < 0.05; **P < 0.01 (one-way ANOVA followed by Games–Howell test).

We previously reported that hepatic cells differentiated from human MSCs in the presence of a Wnt/β-catenin signaling inhibitor could ameliorate acute liver injury upon orthotopic transplantation as cell sheets^21,23^. Although both hexachlorophene and IC-2 induced the hepatic differentiation of MSCs, the effect of IC-2 on this process was more potent than that of hexachlorophene^22,23^. Moreover, IC-2 sheets potently suppressed acute liver injury compared to that with hexachlorophene sheets^23^. In the present study, we clarified the anti-fibrotic effect of IC-2 sheets on liver fibrosis, by comparing it to that of untreated MSC sheets (i.e. MSC sheets). We then generated a model of chronic liver injury using immunodeficient mice for the transplantation of human cells into the liver. Since B cells are indispensable for the establishment of liver fibrosis^24^, we used BALB/c-nu/nu mice for the CCl_4_-induced liver fibrosis model (Fig. 1A). Eleven weeks after the initiation of CCl_4_ administration, mice, which were equally divided into three groups according to liver function tests and body weights, received transplantation of three-layer IC-2 sheets and MSC sheets at two sites of the liver surface (i.e. IC-2 and MSC group, respectively). Sham-operated mice (i.e. sham group) served as a control. The continuous administration of CCl_4_ into three groups of mice was performed for another week, and all mice were sacrificed 9 days after transplantation. Azan staining, Sirius red staining, and immunohistochemistry for type I collagen showed that the IC-2 group exhibited a significant reduction in liver fibrosis compared to that in the sham group (Fig. 1B,C). Moreover, a remarkable reduction in hydroxyproline content was observed in the IC-2 group, compared to that in the sham and MSC group (Fig. 1D). Further, mature type I collagen was prominently decreased in the IC-2 group (Figs 1E, S1A), and only this treatment group exhibited a significant reduction in collagen content compared to that in the sham group. Type III collagen was also decreased following IC-2 sheet transplantation (Fig. S1B). Since the reduction in type III collagen was not as pronounced as that of type I collagen, the reduction in fibril contents seemed to be preferentially affected by the decrease in type I collagen through IC-2 sheet transplantation. These data showed that IC-2-treated cell sheets could consistently mediate a significant reduction in collagen fibril content. Serum alanine transaminase (ALT) was significantly decreased in the IC-2 group, whereas serum aspartate transaminase (AST) and total bilirubin were not changed (Fig. S2A–C). The number of Ki-67-positive hepatocytes was increased in the IC-2 group, and mitotic hepatocytes gradually increased in the sham, MSC, and IC-2 groups in that order (Fig. S2D–F). Improved ALT levels and the promotion of hepatocyte growth were not observed in the MSC group; therefore, MSCs gained both activities through IC-2 treatment. Our data suggest that IC-2 sheets can reduce liver fibrosis and stimulate liver regeneration.

### Hepatic stellate cell activation is suppressed by IC-2 sheet transplantation

**Figure 2:**
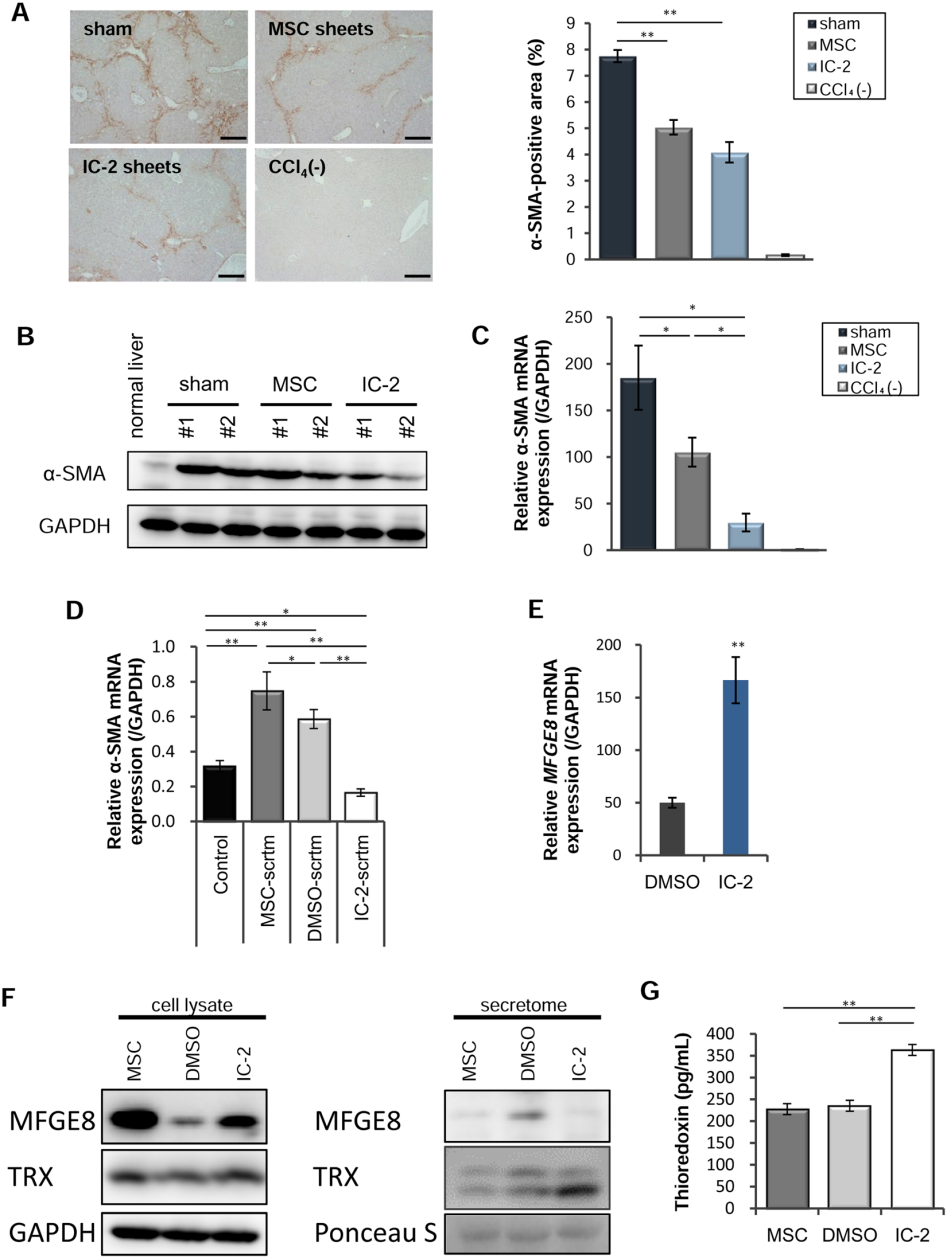
Reduction of hepatic stellate cell activation by IC-2 sheets. (**A**) Immunohistochemistry for alpha-smooth muscle actin (α-SMA) in recipient livers (left). Quantification of α-SMA-positive areas (right; n = 7–9 except for n = 3 for CCl_4_(−) group; CCl_4_(−) indicates control mice without CCl_4_ intoxication; mean ± S.E.M., *P < 0.05, **P < 0.01, one-way ANOVA, Games–Howell test). MSC, mesenchymal stem cell. (**B**) α-SMA expression in the recipient livers was measured by western blotting and (**C**) α*-SMA* expression in the recipient liver was measured by qRT-PCR analysis (n = 6 except for n = 3 for CCl_4_(−) group; mean ± S.E.M., *P < 0.05, one-way ANOVA, Games–Howell test). (**D**) qRT-PCR analysis of α*-SMA* in LX-2 cells after treatment with each secretome harvested from untreated, vehicle-treated, or IC-2-treated MSCs (n = 3, mean ± S.E.M., *P < 0.05, **P < 0.01, one-way ANOVA, least significant difference test). (**E**) *MFGE-8* expression in MSCs on day 7 *in vitro* (n = 3, mean ± S.D., **P < 0.01, Student’s t-test). (**F**) MFGE8 and thioredoxin (TRX) expression in cell lysates (left) and secretomes (right), as analyzed by western blotting. GAPDH and Ponceau S were used as a loading control. (**G**) The secretion of TRX was determined by ELISA (n = 3, mean ± S.D., **P < 0.01, one-way ANOVA, least significant difference test).

Liver fibrosis is characterized by the excessive accumulation of ECM as a result of a disruption in the balance between fibrogenesis and fibrolysis. As such, targeted anti-fibrotic therapies addressing molecules and mechanisms central to fibrogenesis and fibrolysis are required for patients with liver cirrhosis^25^. Fibrogenesis is regulated by activated hepatic stellate cells (HSCs), and is regarded as the main target of anti-fibrotic therapy^1,2,26^. To clarify whether the anti-fibrotic effect of IC-2 sheets was due to decreased fibrogenesis or increased fibrolysis, we examined the status of HSCs. Alpha-smooth muscle actin (α-SMA) is a major indicator of HSC activation. α-SMA-positive areas were markedly decreased in the MSC and IC-2 groups compared to those in the sham group (Figs 2A,B, S3). Moreover, *α-SMA* expression levels in the IC-2 group were decreased compared to those in the MSC group (Fig. 2C). Our results thus suggest that IC-2 sheets can suppress HSC activation in CCl_4_ chronically-administered mice.

Recently, hepatically-differentiated MSCs have been reported to ameliorate liver fibrosis through the secretion of milk-fat globule epidermal growth factor (MFGE)-8^27^. We first examined the effect of IC-2-treated MSC secretomes on HSC activation. The addition of that from IC-2-treated MSCs decreased α-SMA expression in LX-2 human HSCs, suggesting that the secretome of IC-2-treated MSCs contains humoral factors that suppress HSC activation (Fig. 2D). Although MFGE-8 was increased in the cell lysates of MSCs treated with IC-2 (Fig. 2E,F), this marker was not increased in the secretome, contrary to our expectations (Fig. 2F). Thioredoxin (TRX) was increased in both cell lysates and the secretome of MSCs treated with IC-2 (Fig. 2F,G). In our previous report, TRX was upregulated in livers transplanted with hexachlorophene- and IC-2-treated cell sheets^21,23^. Moreover, the upregulation of TRX via knockdown of TRX-interacting protein was shown to suppress HSC activation^28^. In addition, a TRX transgene in mice had a preventive effect on thioacetamide-induced liver fibrosis^29^. These data suggested that TRX is a humoral factor that suppresses HSC activation. Since the activation of these cells was inhibited in both cell sheet-transplanted groups, we investigated whether the resolution of liver fibrosis was due to the indirect effects of cell sheet transplantation via the inhibition of HSC activation. However, the *de novo* expression of several enzymes involved in collagen synthesis and crosslinking, namely collagen 1α1, lysyl oxidase, and prolyl-4-hydroxylase, was not altered in liver tissues (Fig. S4B–D). Furthermore, regarding the *de novo* expression of enzymes involved in the resolution of collagen in recipient mice, matrix metalloproteinase-2 (*Mmp-2*) and *Mmp-8* were decreased in the IC-2 group (Fig. S4E,F), whereas *Mmp-13* and *Mmp-14* remained unchanged (Fig. S4G,H). *Mmp-1a*^30^, a murine homologue of human *MMP-1*, was not detected (data not shown). These data suggest that regarding the *de novo* expression of enzymes involved in the resolution of type I collagen in recipient mice, no functional change in collagen degradation occurred. Our findings suggest that the suppressive effect of IC-2 sheets on liver fibrosis was not due to the *de novo* expression of collagen metabolism-related enzymes in recipient livers.

Next, we examined the possibility that IC-2 itself, included in the cell sheets, inhibits collagen synthesis. The Wnt/β-catenin signaling inhibitor ICG-001 was previously reported to improve liver fibrosis in mice^31^, and its derivative PRI-724 has been used in clinical trials for HCV-related cirrhosis^32^. IC-2 is also a derivative of ICG-001, and therefore, it could be an anti-fibrotic agent. First, we investigated IC-2 content during the preparation of cell sheets. As shown in Fig. S5A, IC-2 content increased with treatment time and reached 478.4 ng/sheet on day 7. Since each mouse received six cell sheets, each animal was exposed to 2.87 µg of IC-2. Assuming that IC-2 is limited to the liver, its concentration was equivalent to 2.3 µM, as the liver volume was determined to be 2.36 ml, calculating this based on an average liver weight of 2.54 g (hepatic volume (in ml) = 0.907 × liver weight (in gram) + 0.053)^33^. Next, we examined whether 2.3 µM of IC-2 would inhibit collagen expression *in vitro*. This concentration was not able to suppress collagen expression in the LX-2 hepatic stellate cell line (Fig. S5B). Therefore, we concluded that the amount of IC-2 included in the cell sheets was not sufficient to reduce liver fibrosis.

### Humoral factors involved in the improvement of liver fibrosis

Since neither the promotion of fibrinolysis nor the suppression of fibrogenesis was observed in recipient livers and because a suppressive effect of IC-2 itself on fibrosis was not observed, the resolution of liver fibrosis by IC-2 sheet transplantation might have been due to the transplanted cell sheets themselves. First, we addressed how the cell sheets affect liver fibrosis based on the distance from the transplanted cell sheets. Focusing on the relationship between the Azan-positive area and the distance from the transplanted cell sheets, improved liver fibrosis was observed in IC-2-treated cell sheet-transplanted mice regardless of the depth from the cell sheets (Fig. S6A). Furthermore, the resolution of liver fibrosis was recognized in another lobe of the liver, where cell sheets were not transplanted (Fig. S6B). Because murine liver lobes are separate from each other, these data support an anti-fibrotic effect of the cell sheets originating from humoral factors via the vascular network. Interestingly, focusing on the relationship between the α-SMA-positive area and the distance from the transplanted cell sheets, the suppression of HSC activation was observed most prominently in mice transplanted with both types of cell sheets beneath the cell sheets (Fig. S6C). These data indicated that the anti-fibrotic effects of the cell sheets and the inhibitory effects of HSC activation are independent mechanisms; specifically, one comprised widespread diffusion to the liver via the host vasculature, whereas the other included the focal penetration beneath the cell sheets.

### Production of MMPs in response to IC-2 treatment

**Figure 3:**
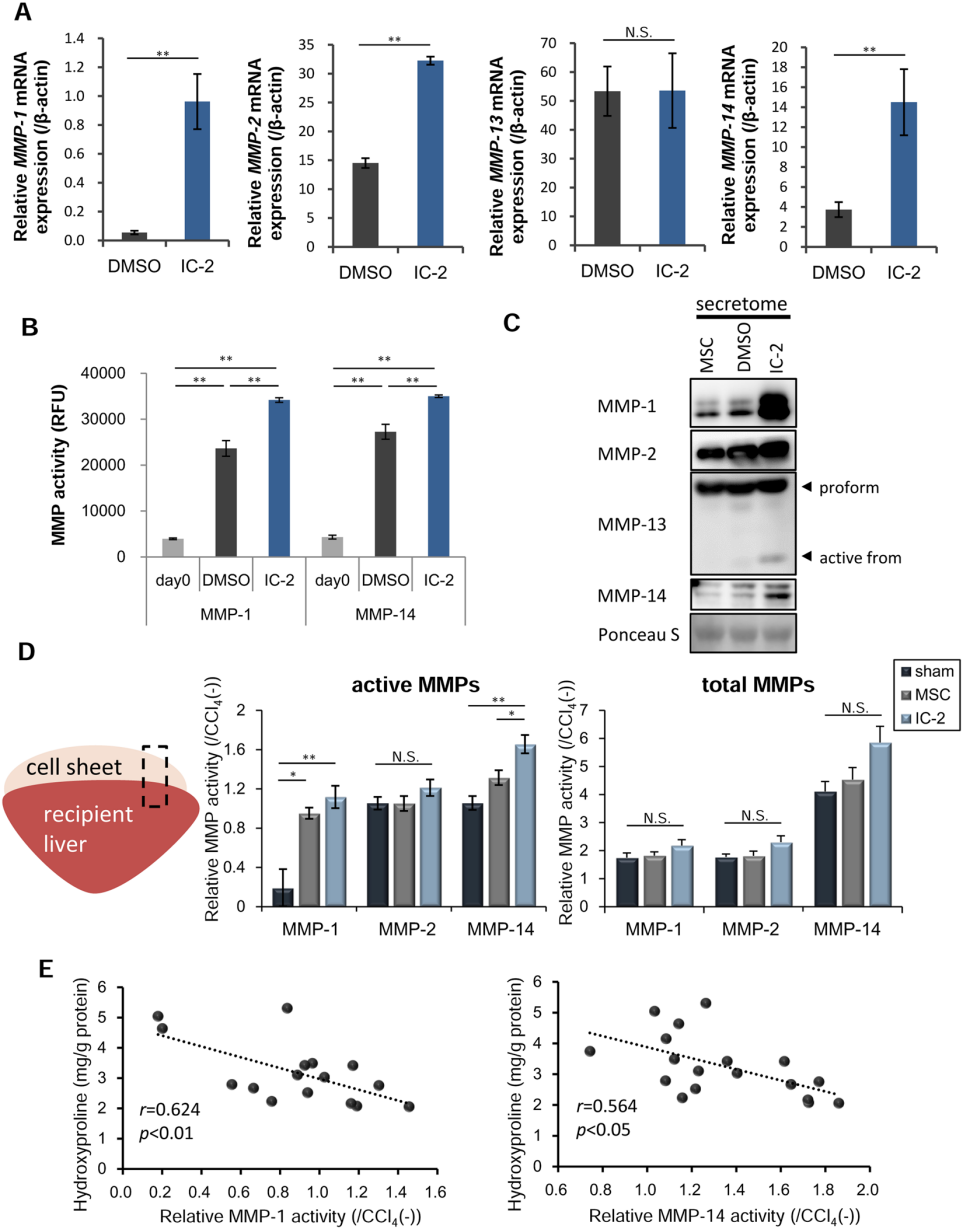
IC-2 increases the production and secretion of matrix metalloproteinases (MMPs) in mesenchymal stem cells (MSCs). (**A**) mRNA expression of fibrolytic genes in MSCs on day 7 *in vitro* (n = 3, mean ± S.D., **P < 0.01, Student’s t-test). (**B**) *In vitro* activities of MMP-1 and MMP-14 in MSCs on day 7 (n = 3, mean ± S.D., **P < 0.01, one-way ANOVA, Tukey test). (**C**) Expression of MMPs in the secretome of MSCs *in vitro*. (**D**) Images of liver tissue used in the experiments and enzymatic activities of MMPs in liver tissues containing cell sheets. Data are expressed as activity relative to that in the CCl_4_(−) group. CCl_4_(−) indicates control mice without CCl_4_ intoxication. Images of liver tissues used in these experiments (left). MMP activities (middle) and activities of total MMPs (right), consisting of active enzyme and latent pro-enzyme (n = 6, mean ± S.E.M., *P < 0.05, **P < 0.01, one-way ANOVA, Games–Howell test). (**E**) Linear regression analysis of relative activities of active MMP-1 (left) or active MMP-14 (right) with respect to hepatic hydroxyproline content 9 days after transplantation (n = 12, Pearson’s correlation coefficient).

Next, we evaluated fibrolytic enzymes that originated from IC-2 sheets. We focused on several MMPs involved in the degradation of type I collagen, which comprises 60~70% of total ECM proteins in cirrhotic livers^34^. Interstitial collagenolytic enzymes (MMP-1, MMP-2, MMP-8, and MMP-13) and membrane type I matrix metalloproteinase (MMP-14) use type I collagen as a substrate^35–37^. IC-2 upregulated the mRNA expression of *MMP-1*, *MMP-*2, and *MMP-*14 in MSCs *in vitro*, whereas *MMP-13* mRNA expression was not altered (Fig. 3A). *MMP-8* mRNA levels were not detected in IC-2-treated MSCs (data not shown). Since *MMP-1*, *MMP-2*, and *MMP-14* mRNA levels were unchanged in hexachlorophene-treated MSCs (data not shown), IC-2 appeared to have additional actions besides inhibition of the Wnt/β-catenin pathway. Among these, MMP-1 and MMP-14 protein levels were increased in IC-2-treated cells, whereas MMP-2 levels were not altered (Fig. S7). Further, the enzymatic activities of MMP-1 and MMP-14 were also increased by IC-2 treatment for 1 week (Fig. 3B). We then examined active forms of the aforementioned MMPs in culture supernatant, since MMP-14 is known to activate both MMP-2 and MMP-13^38,39^. Secretome analysis showed that the active forms of MMP-2 and MMP-13 were increased by IC-2 treatment. In addition to MMP-2 and MMP-13, the secretion of MMP-1 and MMP-14 was also prominently induced by IC-2 (Fig. 3C). Although MMP-14 is known as a membrane-bound type matrix metalloproteinase, the production of a soluble form was also previously reported^40,41^. Taken together, IC-2 enhanced the production of MMP-1 and MMP-14, which are involved in type I collagen degradation.

### MMP-14 is mainly responsible for the resolution of liver fibrosis via IC-2 sheet transplantation

We next investigated which MMPs are involved in the resolution of fibrosis. Liver tissues containing cell sheets from the IC-2 group had higher levels of active MMP-1 and MMP-14 than those of the sham and MSC groups (Fig. 3D). However, significant changes in the activities of total MMPs were not observed among the three groups (Fig. 3D). In contrast, the relative activities of active and total MMP-1, MMP-2, and MMP-14 were not different among sham, MSC, and IC-2 groups in the mouse liver (Fig. S4I,J). Therefore, these data suggest that upregulation of MMP-1 and MMP-14 activities are mediated by transplanted IC-2-treated cell sheets. Hepatic hydroxyproline contents 1 week after transplantation were inversely correlated with relative MMP-1 and MMP-14 activity at same time point (Fig. 3E). These results suggest that MMP-1 and MMP-14 secreted from IC-2 sheets play an important role in the resolution of liver fibrosis. However, as an important problem, these MMP activities were not specific to each substrate. Therefore, we investigated the expression of MMP-1, 2, 3, 7, 8, 9, 12, 13, and 14, during the measurement of MMP activities. As a result, only *MMP-1*, *2*, and *14* were detected in liver tissues containing transplanted cell sheets by RT-PCR analysis (Fig. S8). MMP-2 activities were not altered among the experimental groups (Fig. 3D); therefore, it was important to reveal which MMPs exert the anti-fibrotic effect of IC-2-treated MSC sheets.

**Figure 4:**
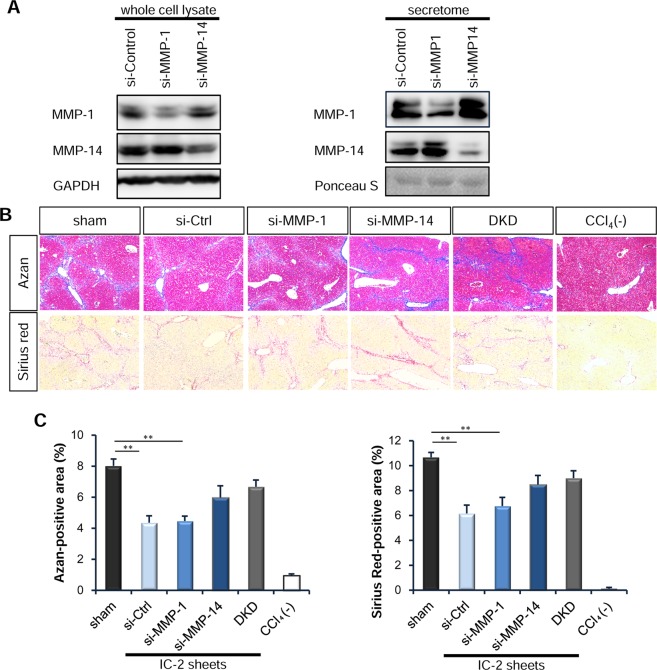
MMP-14 plays an important role in the anti-fibrotic effect of IC-2 sheets. (**A**) Downregulation of MMP-1 or MMP-14 expression through small interfering RNA (siRNA) transfection in IC-2 sheets was confirmed by western blotting. (**B**) Micrographs of Azan (upper) and Sirius red (lower) staining on the sixth day after transplantation in liver tissues transplanted with IC-2 sheets in which transfection of si-MMP-1 and/or si-MMP-14 was performed. (**C**) Measurement of the extent of fibrosis based on Azan staining (left) and Sirius red staining (right) (n = 5–6 per group except for n = 4 for DKD and n = 3 for CCl_4_(−) group. DKD group and CCl_4_(−) group indicate double knock-down of MMP-1 and MMP-14 and control mice without CCl_4_ intoxication, respectively (mean ± S.E.M., **P < 0.01, one-way ANOVA, Games–Howell test).

For this, we knocked down MMP-1 and/or MMP-14 through small interfering RNA (siRNA) transfection during the preparation of IC-2 sheets. Reverse transfection of each siRNA was successfully performed to suppress the expression of MMP-1 and MMP-14 in both cell lysates and secretomes (Fig. 4A). Azan and Sirius red staining were also performed to compare the anti-fibrotic effects on liver fibrosis by transfecting each siRNA into IC-2 sheets. Liver fibrosis was significantly reduced in the group transplanted with si-Ctrl-transfected IC-2 sheets compared to that in the sham group based on Azan staining (Fig. 4B). Transplantation of si-MMP-1-transfected IC-2 sheets achieved almost the same reduction in liver fibrosis as si-Ctrl-transfected IC-2 sheets. However, the anti-fibrotic effect was diminished with IC-2 sheets transfected with si-MMP-14 or both si-MMP-1 and si-MMP-14. A similar result was also obtained based on Sirius red staining (Fig. 4C). These results suggest that the fibrolytic activity of IC-2 sheets on liver fibrosis is dependent on MMP-14. Although MMP-1 possesses potent collagenolytic activity^42^, the fibrinolytic effect of si-MMP-1-transfected IC-2 sheets was marginal. To assess the expression of these MMPs in cell sheets over time, *MMP-1* and *MMP-14* levels were determined in liver tissues containing cell sheets by reverse-transcription polymerase chain reaction (RT-PCR) analysis using human-specific primers (Fig. S9A). One day after transplantation, the expression of *MMP-1* and *MMP-14* was markedly increased in IC-2 sheets and was somewhat diminished in MSC sheets. On 9 day after transplantation, the expression of *MMP-1* became much weaker in both IC-2 and MSC groups. In contrast, the expression of MMP-14 in both groups was stable 9 days after transplantation (Fig. S9A). Furthermore, western blot analysis of the liver tissues containing cell sheets after knockdown experiments showed that MMP-1 protein became undetectable in IC-2 sheets transfected with si-Ctrl 6 days after transplantation. However, the expression of MMP-14 was still apparent in IC-2 sheets transfected with si-Ctrl or si-MMP-1 6 days after transplantation (Fig. S9B). These data suggest that the expression of MMP-1 is diminished at the earlier phase, but that robust expression of MMP-14 was maintained for at least 9 days.

### Primary human mesenchymal stem cell-engineered hepatic cell sheets with IC-2 also suppress liver fibrosis

**Figure 5:**
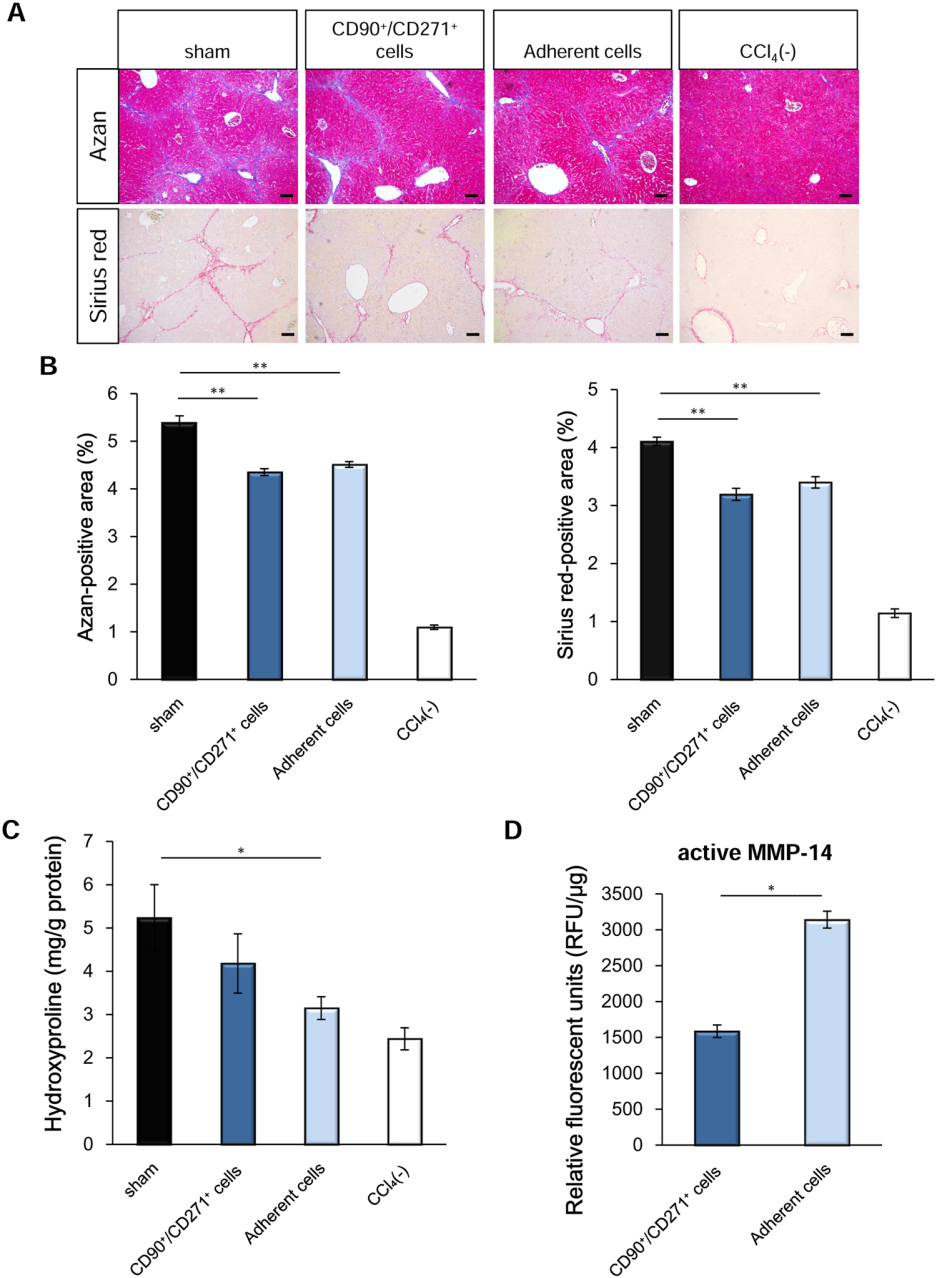
Comparison of the anti-fibrotic effects of IC-2 sheets between CD90^+^/CD271^+^ bone marrow-derived mononuclear cells (BM-MNCs) and adherent BM-MNCs. (**A**) Micrographs of IC-2 liver sections subjected to Azan staining (upper) and Sirius red staining (lower). (**B**) Measurement of fibrotic areas based on Azan and Sirius red staining (n = 9–10 except for n = 3 for CCl_4_(−) group. CCl_4_(−) indicates control mice without CCl_4_ intoxication; mean ± S.E.M., *P < 0.05, one-way ANOVA, least significant difference test). (**C**) Hydroxyproline contents in liver tissues (n = 9–10 except for n = 3 for CCl_4_(−) group, mean ± S.E.M., *P < 0.05, one-way ANOVA, least significant difference test). (**D**) MMP-14 activity in BM-MNCs on the eleventh day after IC-2 treatment *in vitro* (n = 3, mean ± S.D., *P < 0.05, student’s t-test).

Since the aforementioned results were obtained using cell sheets manufactured from UE7T-13 cells, we assessed whether IC-2-engineered primary MSCs are also effective. It was previously reported that bone marrow-derived mononuclear cells that adhere to plastic dishes generally contain MSCs^43^. CD90^+^CD271^+^ bone marrow-derived mononuclear cells have been reported to include MSCs with a high number of colony-forming unit fibroblasts (CFU-Fs) and have elevated differentiation capacity toward a mesenchymal lineage^44^. To investigate which population of MSCs would be suitable for manufacturing IC-2 sheets, we compared the anti-fibrotic effect of IC-2 sheets derived from the two respective cell populations by Azan and Sirius red staining (Fig. 5A). Both cell populations were prepared from bone marrow-derived mononuclear cells obtained from Lonza Inc. Azan-positive areas in mouse livers transplanted with adherent cell-derived cell sheets were significantly decreased compared to those in the sham group (P < 0.01), and those in mouse livers transplanted with CD90^+^CD271^+^ cell-derived sheets were also significantly reduced (P < 0.01, Fig. 5B). Moreover, Sirius red-positive areas in both groups were significantly reduced compared to those in the sham group (Fig. 5B). Hydroxyproline contents in liver tissues transplanted with adherent cell-derived sheets were significantly decreased compared to those in the sham group, but those in CD90^+^CD271^+^ cell-derived sheets were not reduced (Fig. 5C). Furthermore, the activity of MMP-14 in adherent cell-derived sheets was significantly higher than that in CD90^+^CD271^+^ cell-derived sheets (Fig. 5D). These data suggest that adherent cells are a more suitable source than CD90^+^CD271^+^ cells for the treatment of liver fibrosis.

Finally, we examined the effects of the harvesting method and plating cell density on the activities of MMP-1 and MMP-14. With respect to cells treated with IC-2, sheet formation resulted in higher MMP-1 and MMP-14 activity than suspension cells in three individual samples (Fig. S10A,B). Especially, all MSCs showed higher MMP-1 and MMP-14 activities upon sheet formation compared to those with suspension cells. Furthermore, the activities of MMP-1 and MMP-14 in IC-2 sheets were associated with the cell density upon inoculation (Fig. S10C). These data suggest that IC-2 sheets manufactured by plating a higher cell density are suitable for the treatment of liver fibrosis.

## Discussion

### Effects of IC-2-engineered cell sheets on liver fibrosis

Based on our previous findings that MSC-engineered cell sheets produced with Wnt/β-catenin inhibitors could ameliorate acute liver injury^21,23^, we examined the anti-fibrotic effect of IC-2 sheets on CCl_4_-induced liver fibrosis in the present study. Orthotopic transplantation of IC-2 sheets potently reduced liver fibrosis based on Azan staining, Sirius red staining, immunohistochemistry for type I collagen, and hepatic hydroxyproline contents, when compared to that with unmanipulated MSC sheets. The expression of α-SMA indicated that HSC activation was also suppressed by IC-2 sheets. However, the mRNA expression of fibrogenic factors (e.g. collagen 1α1, lysyl oxidase, and prolyl-4-hydroxylase) was not significantly reduced *de novo* in mouse liver tissues. Further, higher activities of MMP-1 and MMP-14 were observed in liver tissues containing IC-2 sheets. In addition, the secretome of IC-2-treated MSCs contained abundant MMP-1 and MMP-14, suggesting that IC-2 sheets secrete these MMPs. Since *MMP-1*, *MMP-2*, and *MMP-14* mRNA levels were not changed in MSCs treated with hexachlorophene, which is a Wnt/β-catenin inhibitor (data not shown), the induction of MMPs was not due to the suppression of this pathway. These data suggest that IC-2 induces MMPs independent of Wnt/β-catenin pathway inhibition.

To our knowledge, there have been no reports thus far indicating that the transplantation of cell sheets can suppress liver fibrosis *in vivo*. MMPs comprise a family of Zn^2+^-dependent endopeptidases^34^; therefore, MMP activity might be inhibited by ethylenediaminetetraacetic acid (EDTA) through the chelation of metal ions^45^. Treatment with trypsin and EDTA can suppress MMP activities, whereas cell sheets enable harvesting without the loss of MMP activities (Fig. S5A,B). MSCs exert potent anti-fibrotic effects through the secretion of MMPs when they are transplanted as cell sheets that are produced in the presence of IC-2. In addition, IC-2 sheets suppress the activation of HSCs possibly through the production of TRX. However, increases in MMPs seem to be the main reason as to why IC-2 sheets potently suppress liver fibrosis. Our findings suggest that MMP-14 is mainly responsible for the improvement of liver fibrosis.

In the present study, the reduction of type I collagen by IC-2 sheets was more efficient than the reduction of type III collagen. It is well known that MMP-14 degrades type III collagen and other ECM components in addition to type I collagen^46^. However, soluble MMP-14, which lacks transmembrane and cytoplasmic domains, has been reported to degrade type I collagen more efficiently than type II or III collagen^47^. Furthermore, the degradation of type I collagen by soluble MMP-14 was also reported to be enhanced synergistically in the presence of MMP-2^47^. In the present study, IC-2 induced MMP-2 expression and secretion in addition to MMP-14 in MSCs (Fig. 3A,C). These data support the fact that type I collagen is more efficiently dissolved by soluble MMP-14 in the presence of MMP-2 produced by IC-2 sheets. Further type I collagen is predominant in the human cirrhotic liver^34^; therefore, IC-2-treated MSC sheets have potential applications for human cirrhosis.

### Cell sheet technology as regenerative medicine for liver fibrosis

Tissue engineering-based cell sheet therapy is suitable for the efficient transplantation of hepatocytes or differentiated hepatic cells. In the present study, we demonstrated that the formation of cell sheets is important to increase the activities of MMP-1 and MMP-14 (Fig. S5A,B). These findings suggest that cell sheet technology is especially useful for the treatment of liver fibrosis, in addition to other advantages such as preserved cellular communication junctions, endogenous extracellular matrix, and integrative adhesive agents^18^. Recently, several groups including us revealed that cell sheet transplantation is useful to ameliorate acute liver failure^21,23,48,49^. In one study using iPSC-derived hepatocyte sheets, it was determined that the therapeutic effects of cell sheets on acute liver injury are due to hepatocyte growth factor (HGF) production^48^. This paper implied that iPSC-derived cell sheets contain hepatic non-parenchymal cells since these cells, but not hepatic parenchymal cells, produce HGF. The transplantation of hepatocytes into radiation-induced or partially hepatectomized livers also resulted in compensated liver functions^49^. Taken together, the combination of cell sheet-based technology with a single small molecule compound such as IC-2 can accelerate the resolution of liver fibrosis. However, if the etiology of cirrhosis continues, repeat transplantation would be required. Repeating cell sheet transplantation in the case of laparotomy is thought to be possible, but it is better to develop the laparoscopic transplantation of cell sheets in the future to reduce the burden on patients that is associated with the use of devices, as occurs with endoscopic cell sheet transplantation^50^.

In summary, IC-2 sheets possess potent anti-fibrotic activity via the induction of MMPs and the suppression of HSC activation. A novel anti-fibrotic therapy based on cell sheet technology will be a potent option for liver fibrosis in the future.

## Methods

### Chemical compounds and cells

IC-2^22,23^, a derivative of ICG-001, was synthesized in house and dissolved in dimethyl sulfoxide (DMSO). The final concentration of DMSO was 0.1%. UE7T-13 human bone marrow-derived MSCs^51^ were used. For experiments using bone marrow mononuclear cells adherent to culture dishes and CD90^+^/CD271^+^ cells, human bone marrow mononuclear cells were purchased from Lonza Inc. (Walkersville, MD) and were used at passage five as previously described^22^.

### Preparation of cell sheets

IC-2-treated cell sheets were manufactured as follows. UE7T-13 cells were plated on ϕ 60-mm temperature-responsive culture dishes (CellSeed Inc., Tokyo, Japan) at a cell density of 9.0 × 10^3^ cells/cm^2^, treated with 15 μM IC-2 for 1 week, and used as IC-2 sheets. MSC sheets were prepared by plating UE7T-13 cells at a density of 1.8 × 10^4^ cells/cm^2^, cultured without IC-2 for 4 days, and used as MSC sheets. One day before transplantation, both cell sheets were detached from ϕ 60-mm temperature-responsive culture dishes by incubating them at 20 °C for ~30 min and then at 20 °C until use.

Adherent cells were harvested from bone marrow-derived mononuclear cells from Lonza, Inc. attached to culture dishes. CD90^+^/CD271^+^ cells were sorted from bone marrow-derived mononuclear cells from Lonza Inc. with a cell sorter using anti-CD90/anti-CD271 antibodies as previously described^22^. Cell sheets were prepared as follows; both cell types were plated on ϕ 60-mm temperature-responsive culture dishes at a cell density of 1.8 × 10^4^ cells/cm^2^ and treated with 30 μM IC-2 for 11 days. Cell sheets were harvested from thermoresponsive polymer-coated culture dishes with CellShifter (CellSeed Inc.), which were overlaid with a ϕ 30-mm support membrane, by incubating them at 20 °C for ~3 h before transplantation.

### siRNA-transfected cell sheet transplantation

For siRNA-transfected cell sheet transplantation, cell sheets were created as follows. UE7T-13 cells were plated onto culture dishes at a density of 9.0 × 10^3^ cells/cm^2^ and treated with 15 μM IC-2 for 7 days. Culture media were replaced 4 days after seeding. Subsequently, 9.64 × 10^6^ IC-2-treated cells were reverse transfected with 600 pmol siRNA using the Lipofectamine RNAiMAX reagent (Thermo Fisher Scientific Inc., MA), and reseeded onto ϕ 100-mm temperature-responsive culture dishes 1 day before harvesting. Six hours after reverse transfection, cells were treated with 15 μM IC-2. Silencer® select validated siRNA (s8879 for si-MMP14, s8849 for si-MMP1, and negative control no.1 siRNA for si-control) were purchased from Thermo Fisher Scientific Inc. siRNA-transfected cell sheets were detached from ϕ 100-mm temperature-responsive culture dishes by incubating them at 20 °C for ~15 min 1 day before transplantation and were incubated at 20 °C until use.

### Cell sheet transplantation and biochemical tests

All animal experiments were conducted in accordance with the ethical approval of the Tottori University Subcommittee on Laboratory Animal Care. All mice were housed under pathogen-free conditions in a temperature-controlled, illuminated (12-h daily) room with *ad libitum* access to water and chow.

To induce chronic liver injury, carbon tetrachloride (CCl_4_) dissolved in olive oil (Wako Pure Chemical Industries Ltd., Osaka, Japan) was orally administered twice per week to 7–9-week-old BALB/c-nu/nu male mice (CLEA Japan, Inc., Tokyo, Japan) at a dose of 0.6 mL/kg for 4 weeks and a dose of 1.2 mL/kg for 6 to 7 weeks. One day before transplantation, the mice were subjected to liver function tests (e.g. serum ALT, AST, and total bilirubin) and their body weights were measured. Mice were equally divided into three groups according to liver function tests and body weight. Transplantation was performed 2 days after the last CCl_4_ dose.

In most cases, three-layer cell sheets were transplanted at two sites on the left lateral lobe. For experiments using adherent cells and CD90^+^/CD271^+^ cells, three-layer, half-sized cell sheets were transplanted at two sites on the left lateral lobe and middle lateral lobe. For siRNA-transfection experiments, two-layer cell sheets were transplanted at one site on the left lateral lobe. CCl_4_ administration was continued for another week after transplantation.

Two days after the final CCl_4_ dose, mice were sacrificed by exsanguination under anesthesia with pentobarbital sodium and blood samples were collected from the inferior vena cava, which was followed by liver resection. Mice transplanted with siRNA-transfected cell sheets were sacrificed 6 days after transplantation. A portion of the liver was fixed in 4% paraformaldehyde and embedded in paraffin for histological analysis. The remaining liver tissues were snap-frozen in liquid nitrogen.

Blood samples were maintained overnight on ice and serum was isolated by centrifugation at 2,000 × *g* for 20 min. Serum aminotransferase and total bilirubin levels were measured as previously reported^21^.

Details of other experimental protocols are provided in the Supplemental Information.

## Supplementary information

Supplementary information
