# Supplementary material for: Reversal of established liver fibrosis by IC-2-engineered mesenchymal stem cell sheets
Source: Sci Rep. 2019 May 2;9:6841. doi: 10.1038/s41598-019-43298-0 (PMC6497888; doi:10.1038/s41598-019-43298-0)
Supplement: Supplementary file 1 — Supplementary information [file 41598_2019_43298_MOESM1_ESM.docx]

Supplementary information

Reversal of established liver fibrosis by IC-2-engineered mesenchymal stem cell sheets Noriko Itaba, Yohei Kono, Kaori Watanabe, Tsuyoshi Yokobata, Hiroyuki Oka, Mitsuhiko Osaki, Hiroki Kakuta, Minoru Morimoto, Goshi Shiota


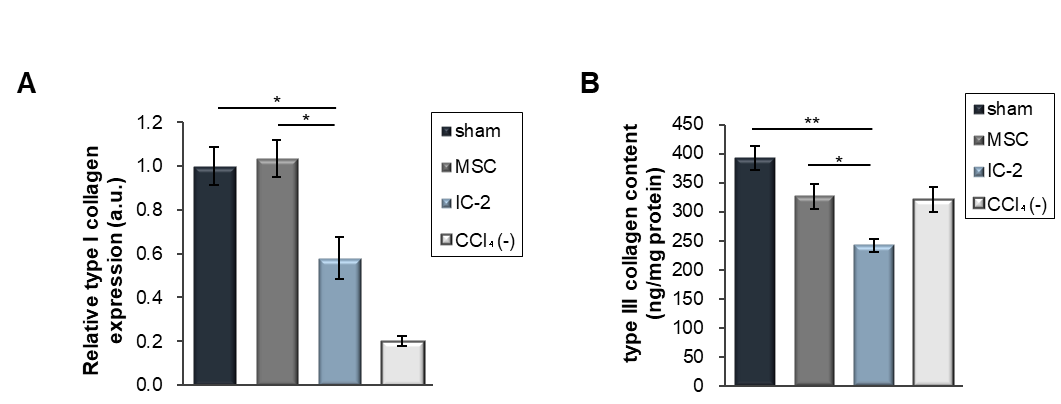


**Figure S1. Orthotopic transplantation of IC-2 sheets reduced collagen content.**

(A) Semi-quantitative analysis of western blotting for type I collagen. Collagen expression was normalized with the same blot of GAPDH, and the mean of expression of each mice was calculated from three independent western blotting analysis (n = 7-8 except for n = 3 for CCl_4_(−)). The results are expressed as the mean ± S.E.M. One-way ANOVA followed by Games–Howell test was used for statistical evaluation. **P* < 0.05. a.u.: arbitrary unit. (B) Hepatic type III collagen content was measured by ELISA. Type III collagen content in each subject was corrected with each protein concentration (n = 7-8 for each group except for n = 3 for CCl_4_(−)). The results are expressed as the mean ± S.E.M. One-way ANOVA followed by Games–Howell test was used for statistical evaluation. **P* < 0.05, ***P* < 0.01.

**
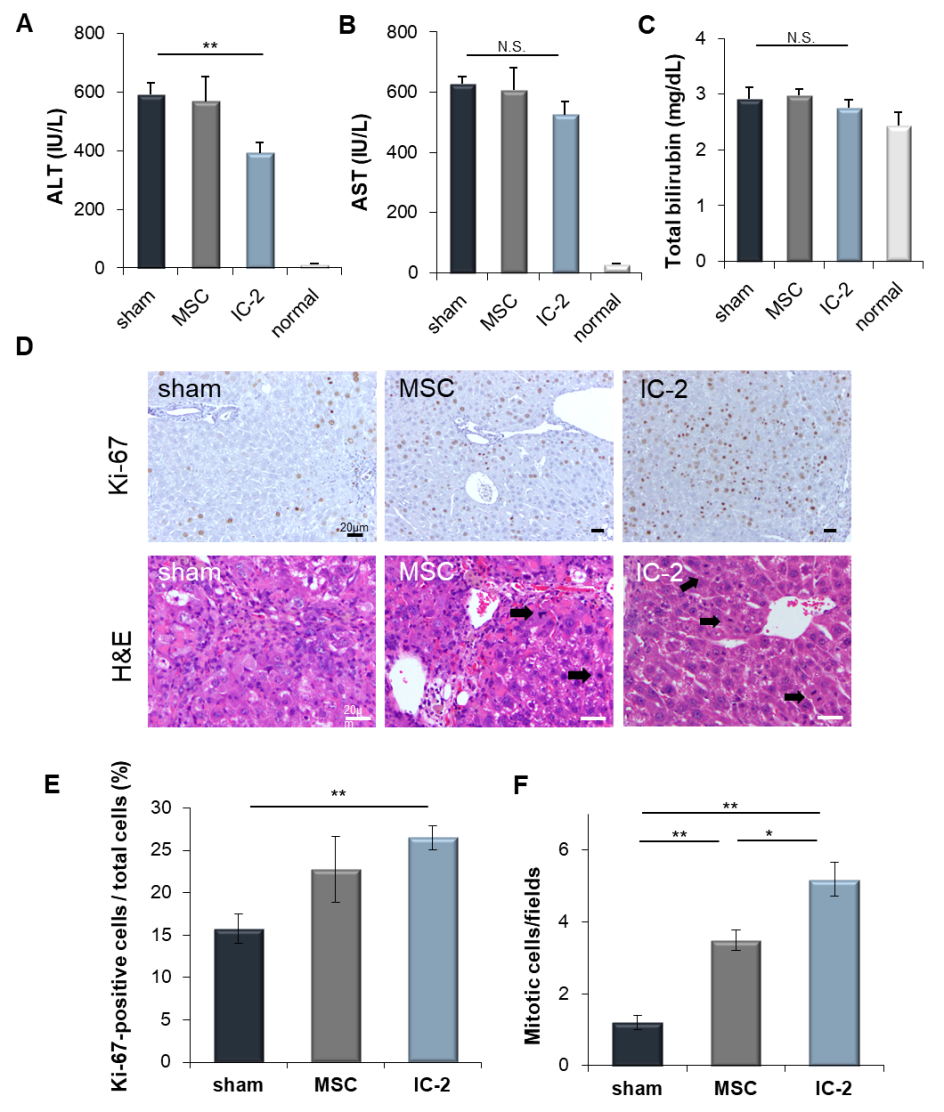
**

**Figure S2.** **Orthotopic transplantation of IC-2 sheets improves liver function and promotes liver regeneration 9 days after transplantation.**

(A-C) Serum ALT (A), AST (B), and total bilirubin (C) of mice (n = 7-9 except for n = 3 for CCl_4_(−)). (D) Micrograph of liver sections stained by immunohistochemistry using anti-Ki-67 antibody (upper) and H&E staining (lower). Mitotic hepatocytes are shown as arrows. (E, F) The numbers of Ki-67-positive cells (E) and mitotic hepatocytes (F) (n = 6 except for n = 3 for CCl_4_(−)). The results are expressed as the mean ± S.E.M. One-way ANOVA followed by Games–Howell test was used for statistical evaluation. **P* < 0.05, ***P* < 0.01.


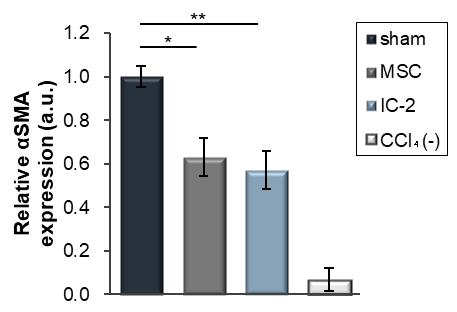


**Figure S3. Orthotopic transplantation of MSC sheets and IC-2-treated MSC sheets inhibited hepatic stellate cells activation.**

Semi-quantitative analysis of western blotting for αSMA. αSMA expression was normalized with the same blot of GAPDH, and the mean of expression of each mice was calculated from three independent western blotting analysis (n = 7-8 except for n = 3 for CCl_4_(−)). The results are expressed as the mean ± S.E.M. One-way ANOVA followed by Games–Howell test was used for statistical evaluation. **P* < 0.05, ***P* < 0.01. a.u.: arbitrary unit.


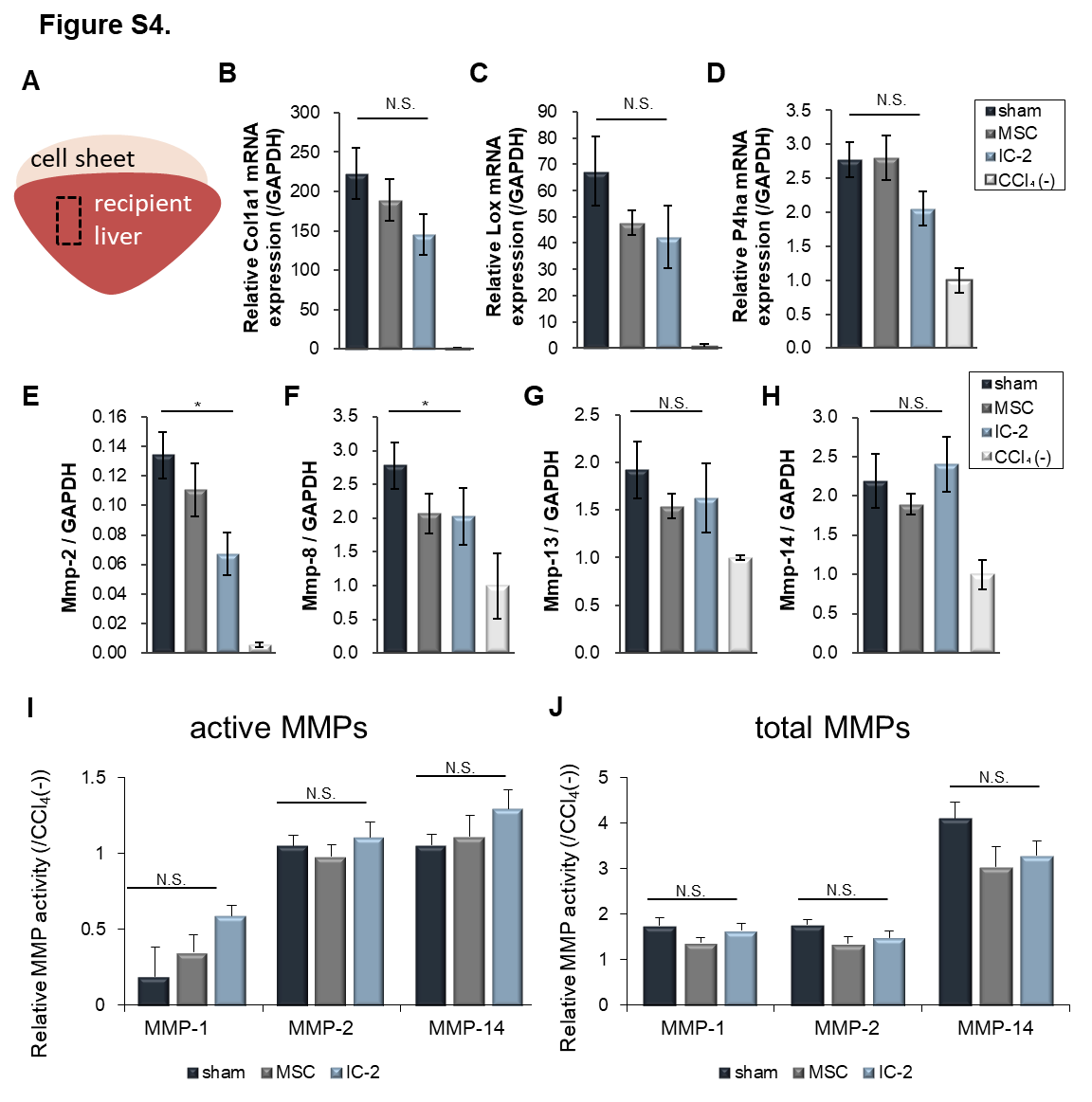


**Figure S4. De novo expression of** **fibrogenic genes and fibrinolytic genes and matrix metalloproteinase (MMP) activity in naiive liver tissues excluding cell sheets.**

(A) Images for liver tissue used in the experiments (B-J). (B-H) mRNA expression of fibrogenic genes (B-D) and fibrinolytic genes (E-H) in recipient liver tissues. (I and J) The relative activities of MMP-1, MMP-2, and MMP-14 in naiive liver tissues. Relative activity was expressed as activity compared to the CCl_4_(−) group. Active MMP activities (I) and total MMP activities composed of active enzyme and latent pro-enzyme (J) were measured. The results are expressed as the mean ± S.E.M (n = 6 except for n = 3 for CCl_4_(−)). One-way ANOVA followed by Games–Howell test was used for statistical evaluation.


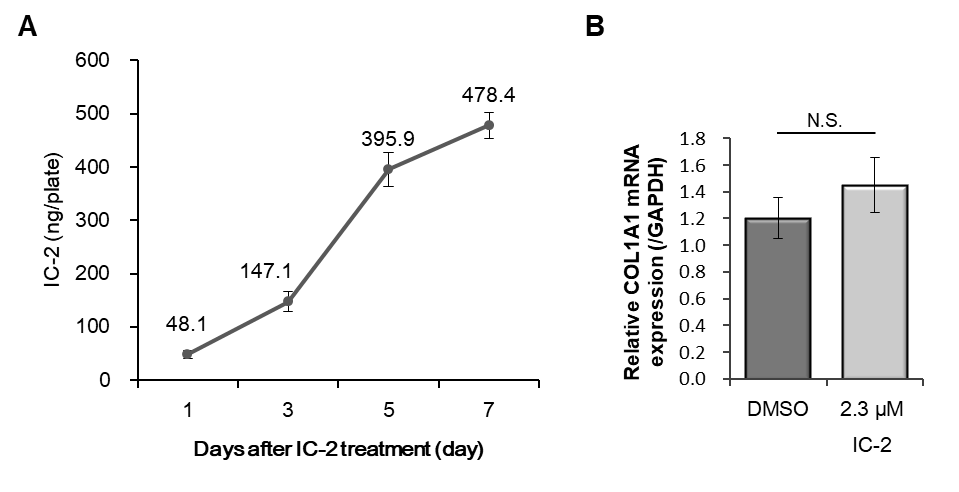


**Figure S5. Examination for inhibitory effects on collagen expression of IC-2 itself in LX-2 HSCs.**

(A) Transition of IC-2 contents during the IC-2 treated MSC sheets preparation was measured by LC/MS (n=3). 478.4 ng of IC-2 was included in a cell sheet. (B) qRT-PCR analysis of COL1A1 mRNA in LX-2 cells after IC-2 treatment equivalent dose included in six cell sheets toward liver volume (n=3). Student’s t- test was used for statistical evaluation.


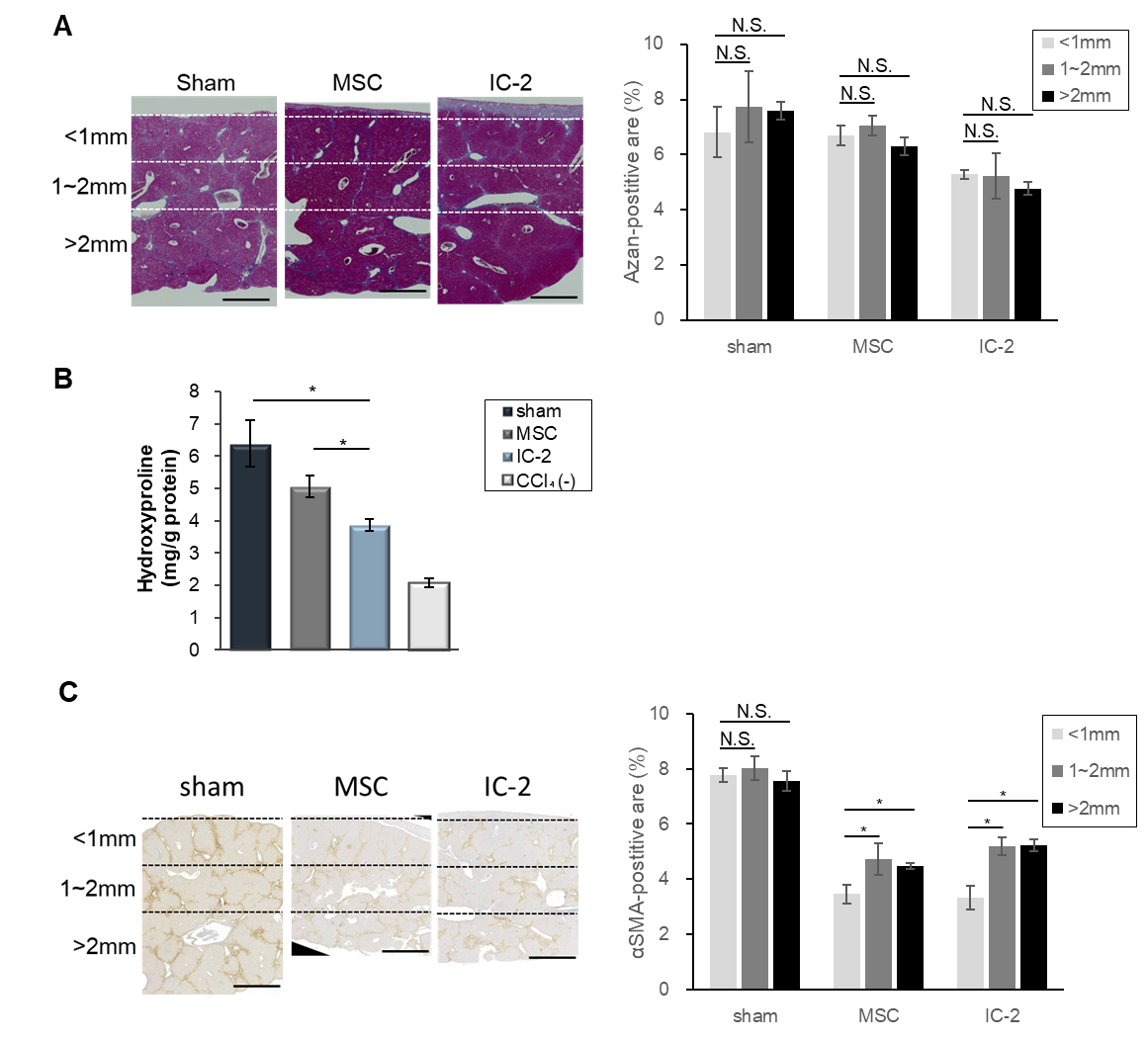


**Figure S6. Relationship between the inhibitory effects of transplanted cell sheet on collagen fiber or HSCs activation and distance from the transplanted cell sheets.**

(A) Relationship between the Azan-positive area and distance from the transplanted cell sheets. The distance from the cell sheets is divided to 3 locations according to their depth from the cell sheets. Representative micrographs of liver sections subjected to Azan staining (left). Scale bar = 1mm. Azan-positive area of each divided area (right). Each percentage depicted the rate of Azan-positive area (n=5). Steel test was used for statistical evaluation. (B) Hydroxyproline contents of the right medial lobe of liver (n = 7 - 8 except for n = 3 for CCl_4_(−)). One-way ANOVA followed by Games–Howell test was used for statistical evaluation. (C) Relationship between the αSMA-positive area and distance from the transplanted cell sheets. Representative micrographs of liver sections subjected to immunohistochemistry for αSMA (left). Scale bar = 1mm. αSMA -positive area of each divided area (right). Each percentage depicted the rate ofαSMA-positive area (n=5). Levels of significance: *P < 0.05 (Steel test).

The results are expressed as the mean ± S.E.M. Levels of significance: *P < 0.05 (One-way ANOVA followed by Games–Howell test).

**
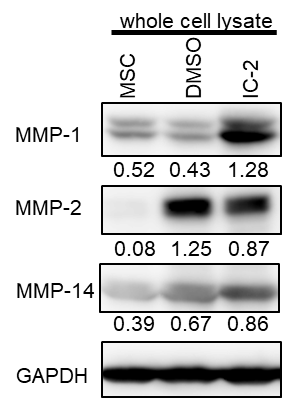
**

**Figure S7. MMP-1, MMP-2, and MMP-14 expression in cell sheets 1 day after peeling from the dish.**

MSC, DMSO, and IC-2 show MSC cell sheets incubated without DMSO, with 0.1% DMSO, and with 15 µM IC-2 in 0.1% DMSO, respectively. The number below the band of each MMP shows the relative expression levels of each MMP compared to GAPDH. Relative expression is calculated by taking the intensity of chemiluminescent reactions.


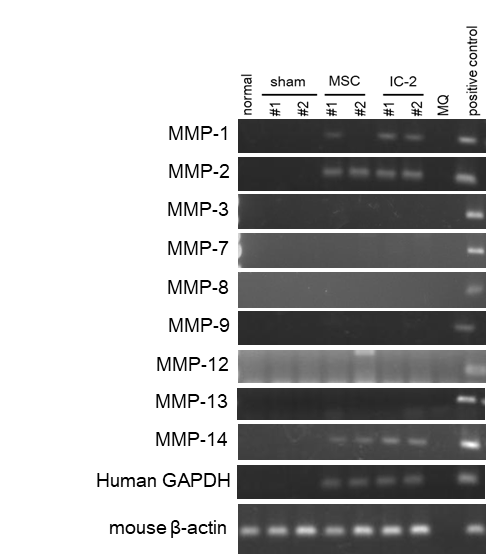


**Figure S8. RT-PCR analysis of specifically recognizing human MMPs expression in the liver tissues including transplanted cell sheets.**

RT-PCR analysis was conducted using primers specifically annealing human mRNA transcripts except for mouse Actb. Positive controls for each gene were described in Figure S18 with full length gels.


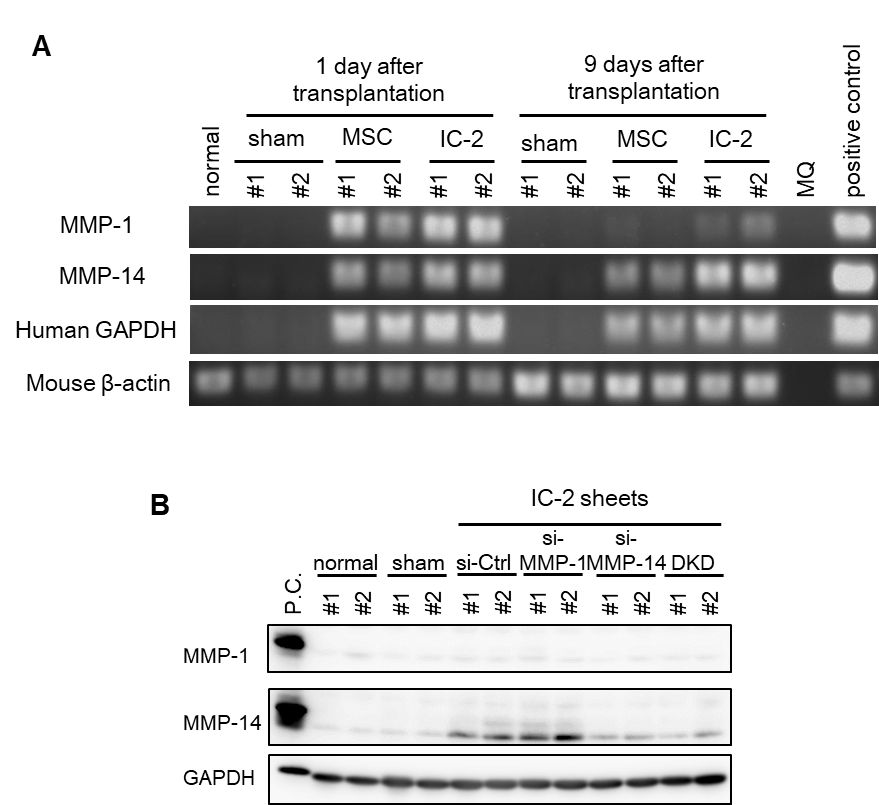


**Figure S9. MMP-1 and MMP-14 expression in liver tissues containing cell sheets.**

(A) RT-PCR analysis detects cell sheet-derived MMP-1 and MMP-14 mRNA using human-specific gene expression-detectable primers. UE7T-13 cDNA and LX-2 cDNA were used as positive controls for MMP-1 and MMP-14, respectively. UE7T-13 cDNA and mouse liver cDNA were used as positive controls for human GAPDH and mouse β-actin, respectively. (B) Western blot of MMP-1 and MMP-14 protein in liver tissues transplanted with siRNA-transfected cell sheets 6 days after transplantation. DKD group indicates double knock-downed of MMP-1 and MMP-14 sheets transplanted mice. DKD: double-knockdown mice.


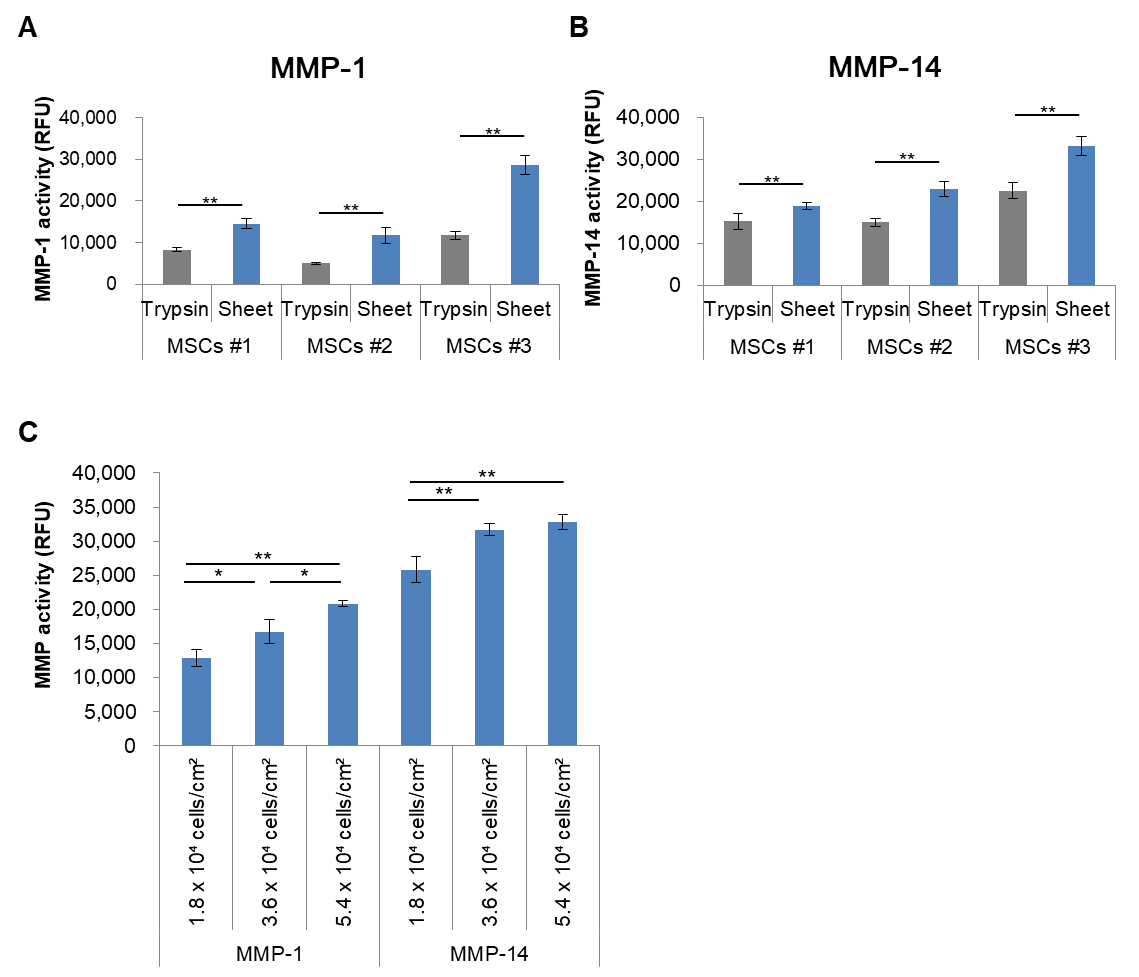


**Figure S10. Activities of MMP-1 and MMP-14 regarding cell sheet formation and cell density.**

(A, B) Activities of MMP-1 and MMP-14 in IC-2-treated adherent MSCs. Cells were harvested under the cell sheet condition or from trypsinized single cells. Adherent MSCs prepared from three independent donors were assessed (n = 3, mean ± S.D., ***P* < 0.01, two-tailed Student’s t-test). (C) Activities of MMP-1 and MMP-14 in IC-2 sheets 7 days after IC-2 treatment in three cell density conditions (n = 3, mean ± S.D., **P* < 0.05, ***P* < 0.01, one-way ANOVA followed by tukey test).


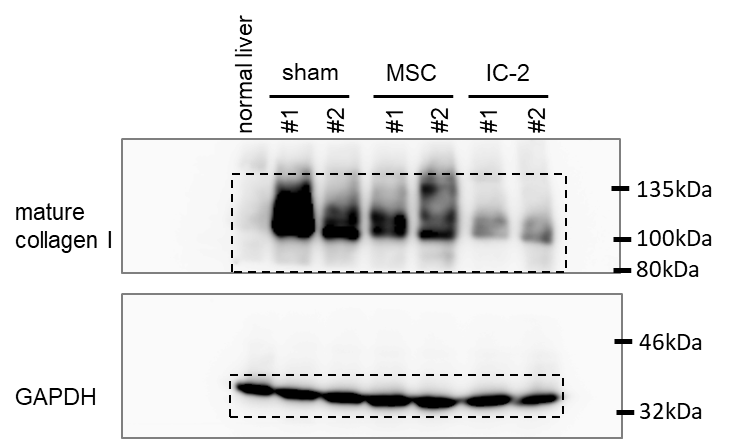


**Figure S11. Full length blots of Figure 1E.** Black dotted lines show the cropping locations. Brightness and contrast were not changed during processing these blots.

**
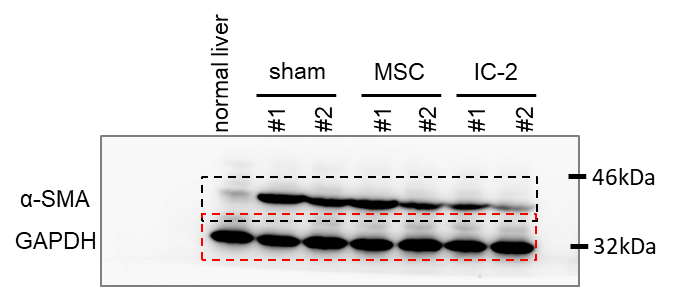
**

**Figure S12. Full length blots of Figure 2B.** Black dotted line (αSMA) and red dotted line (GAPDH) show the eahc cropping location. αSMA and GAPDH were detected in same blot. Brightness and contrast were not changed during processing these blots.


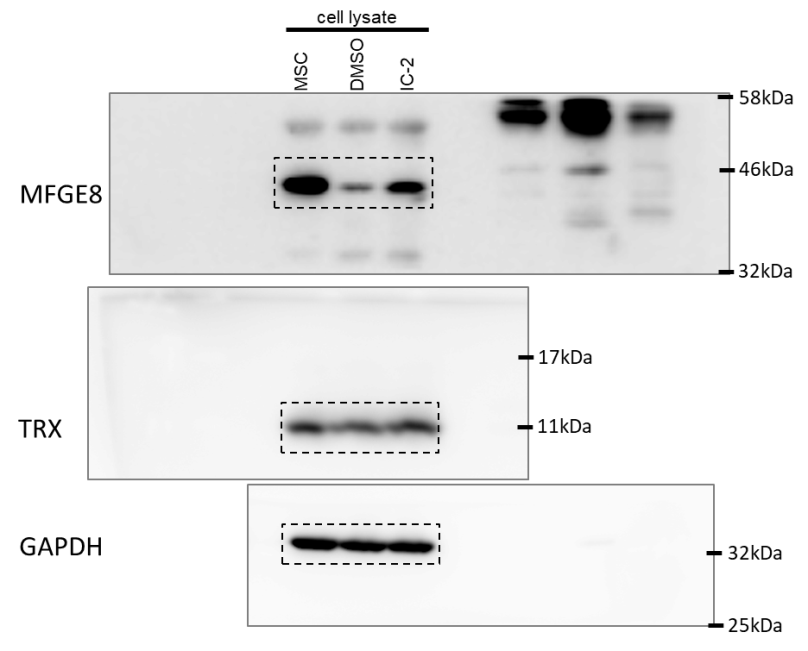


**Figure S13. Full length blots of Figure 2E.** Black dotted lines show the cropping locations. Brightness and contrast were not changed during processing these blots.

**
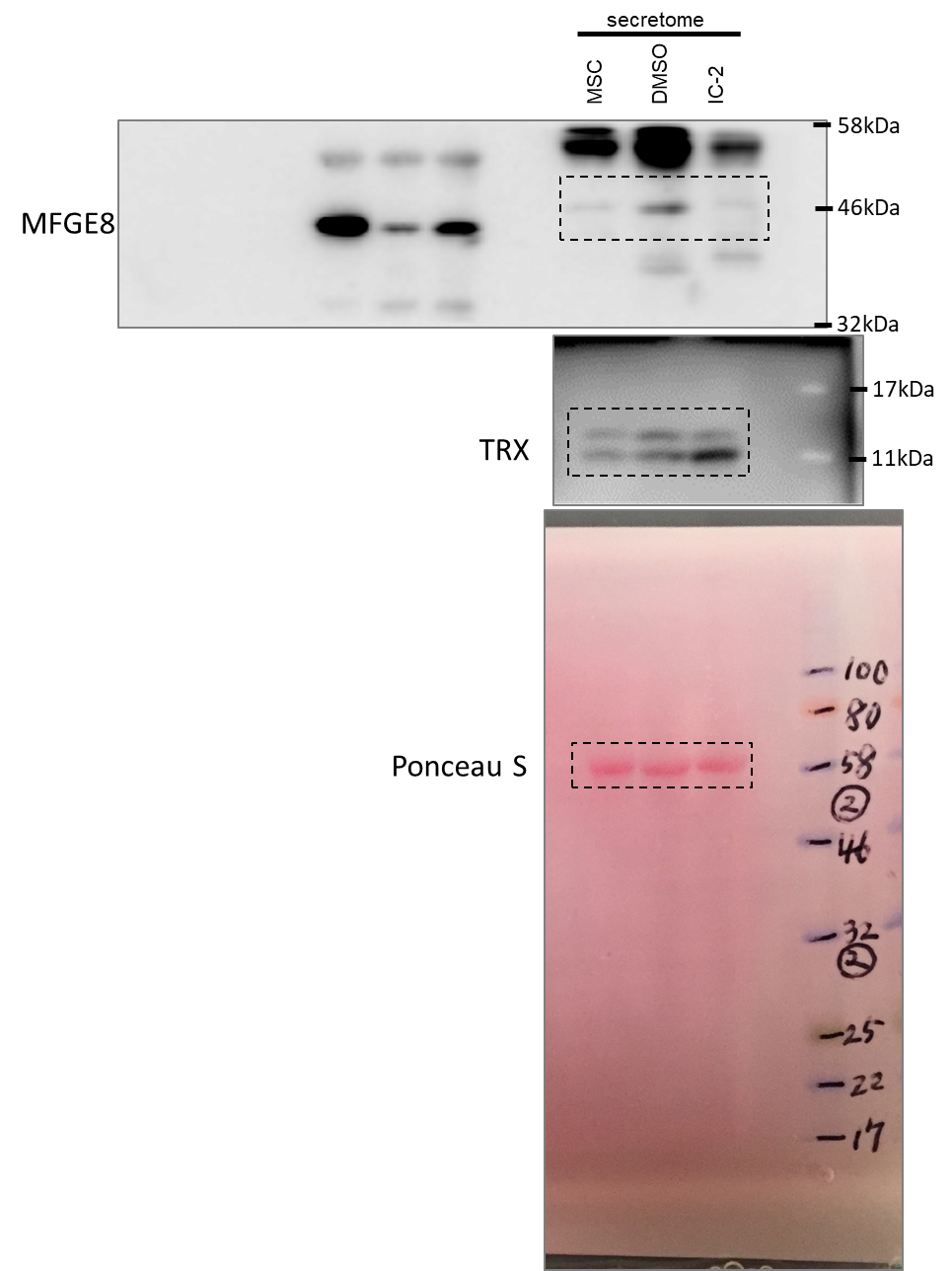
**

**Figure S14. Full length blots of Figure 2E.** Black dotted lines show the cropping locations. Brightness and contrast were not changed during processing these blots. Color image of Poceau S staining was converted to monochrome image.

**
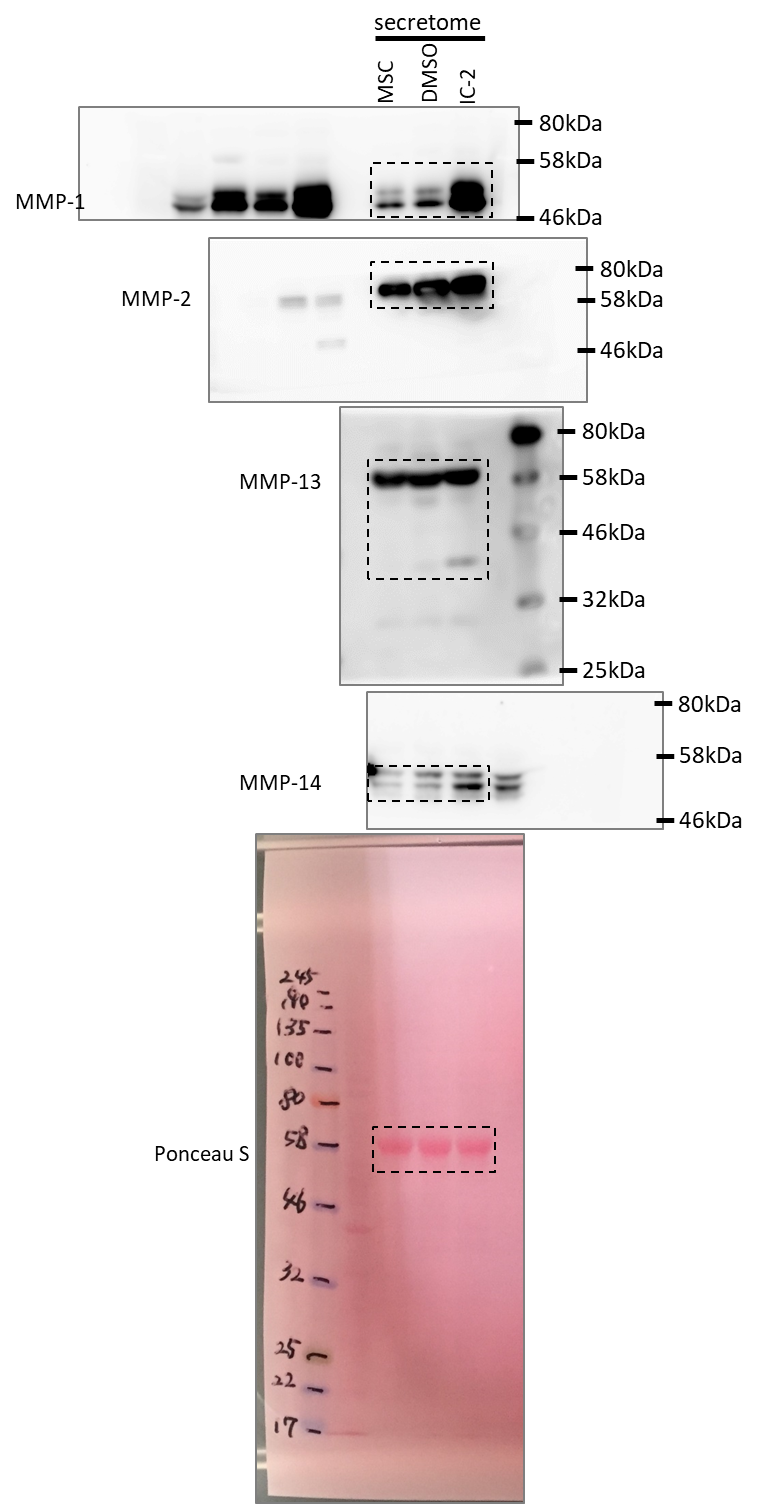
**

**Figure S15. Full length blots of Figure 3C.** Black dotted lines show the cropping locations. Brightness and contrast were not changed during processing these blots. Color image of Poceau S staining was converted to monochrome image.

**
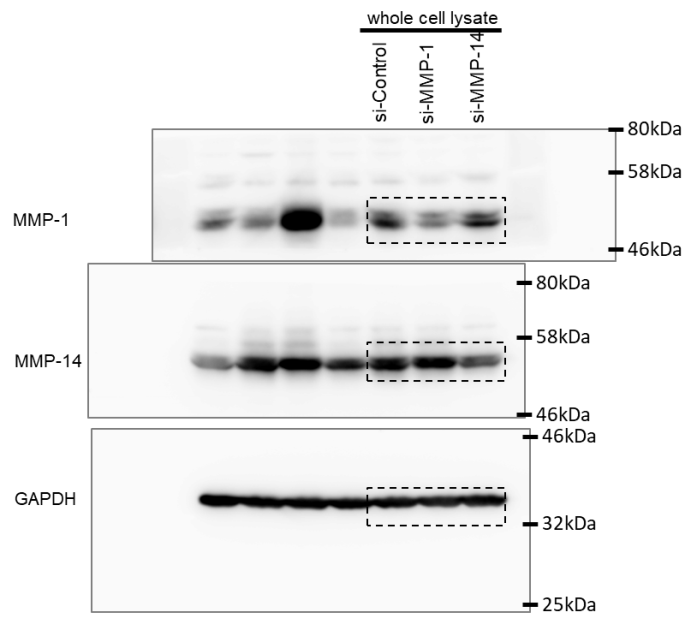

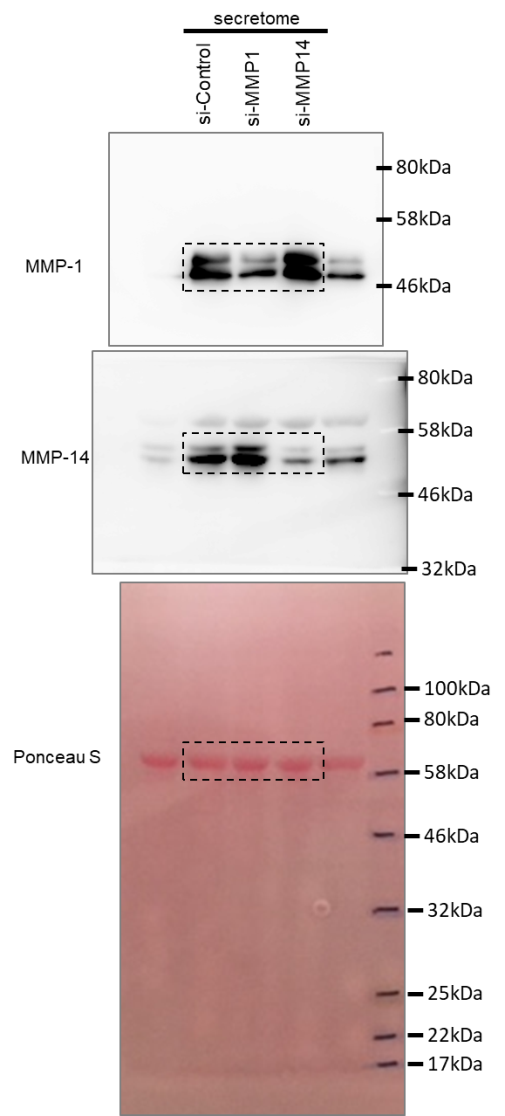
**

**Figure S16. Full length blots of Figure 4A.** Black dotted lines show the cropping locations. Brightness and contrast were not changed during processing these blots. Color image of Poceau S staining was converted to monochrome image.

**
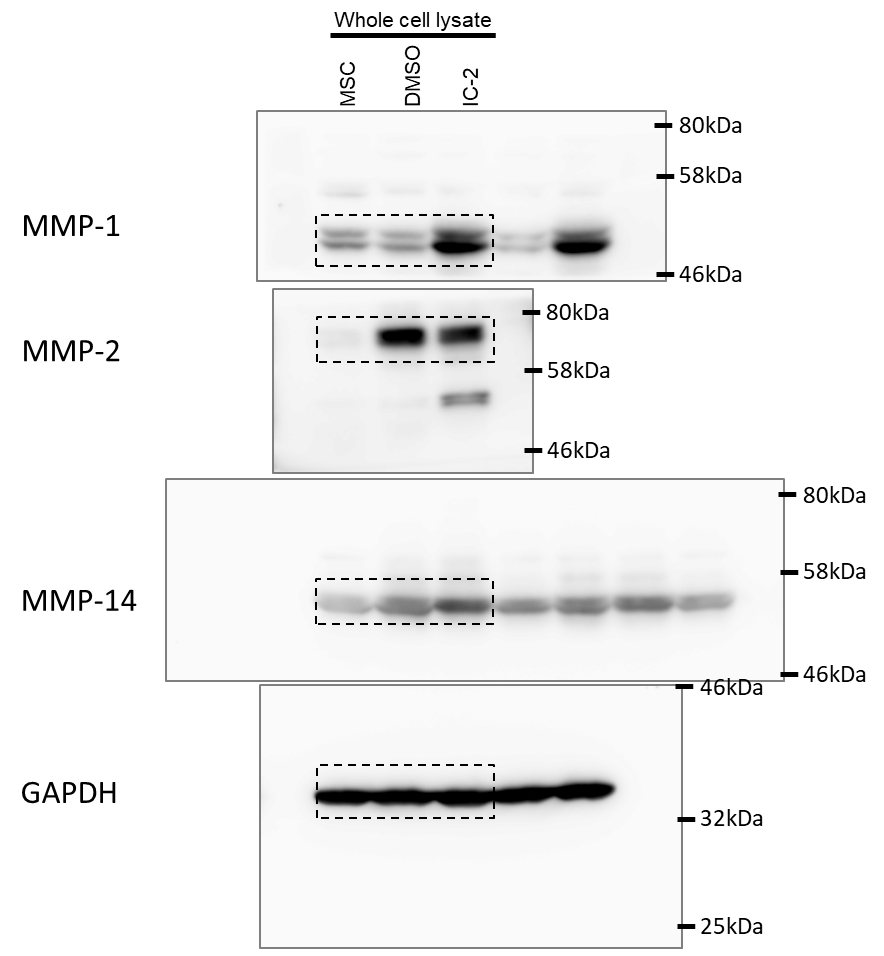
**

**Figure S17. Full length blots of Figure S7.** Black dotted lines show the cropping locations. Brightness and contrast were not changed during processing these blots.

**
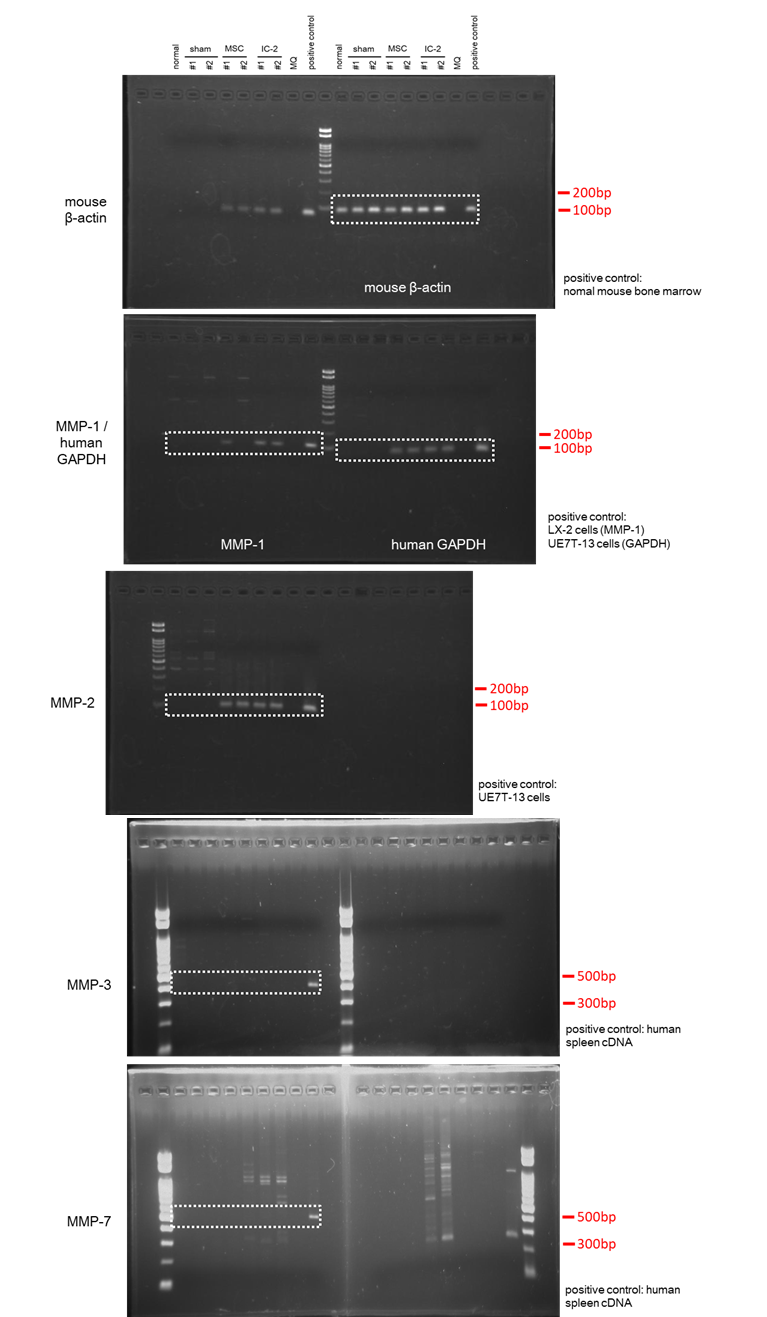
**

**
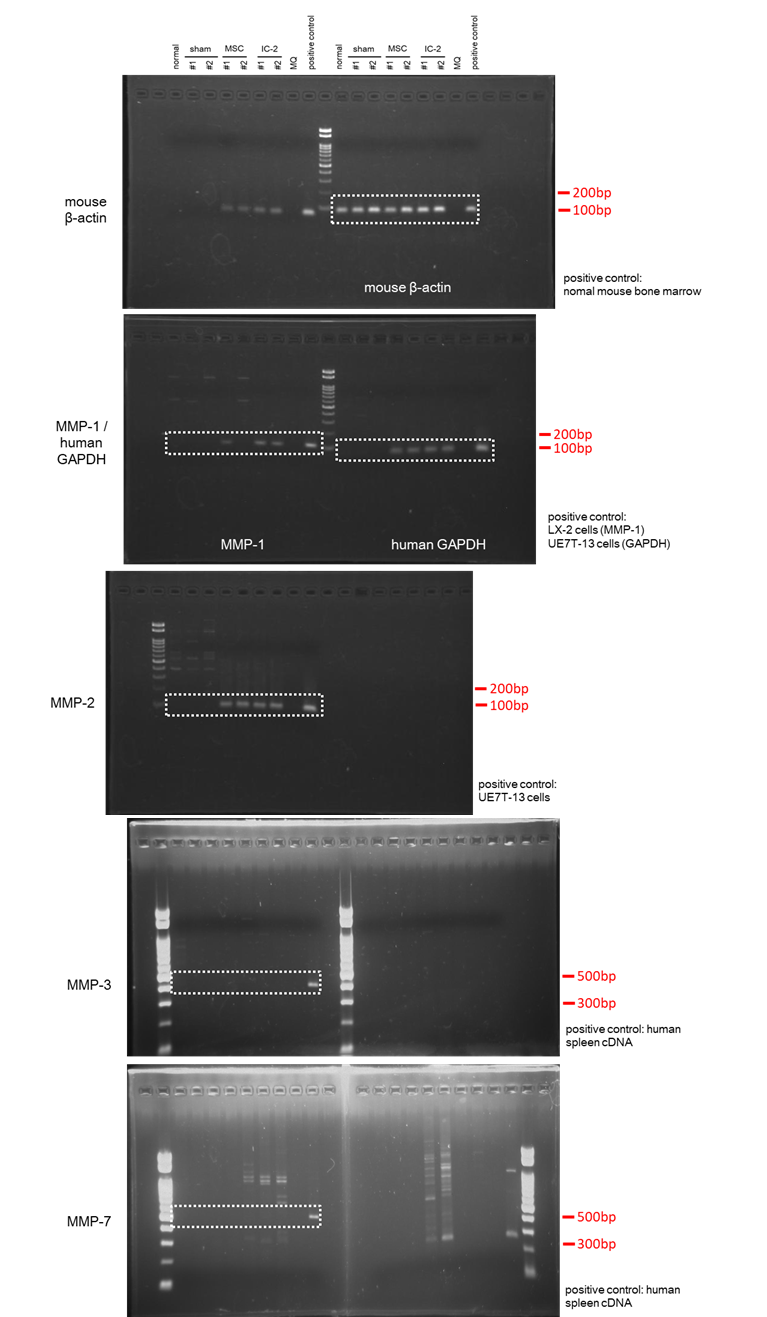

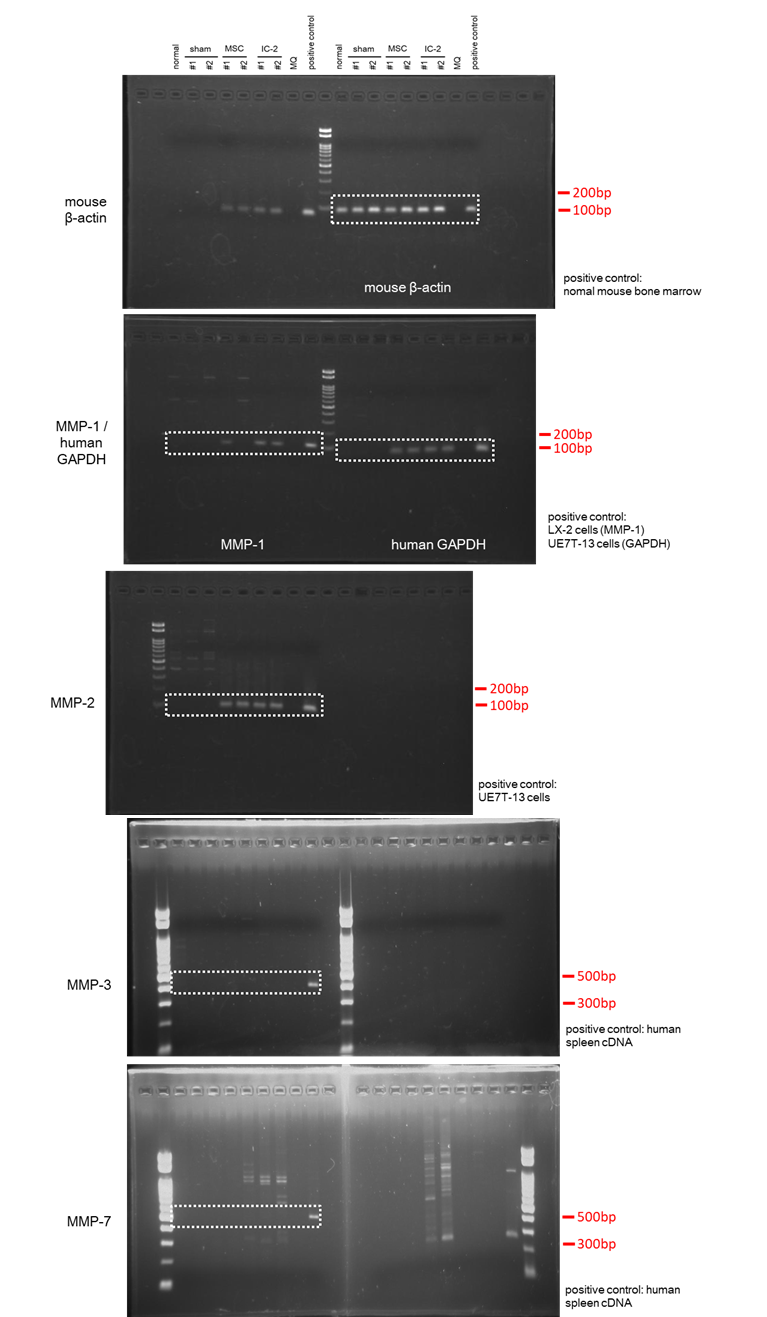

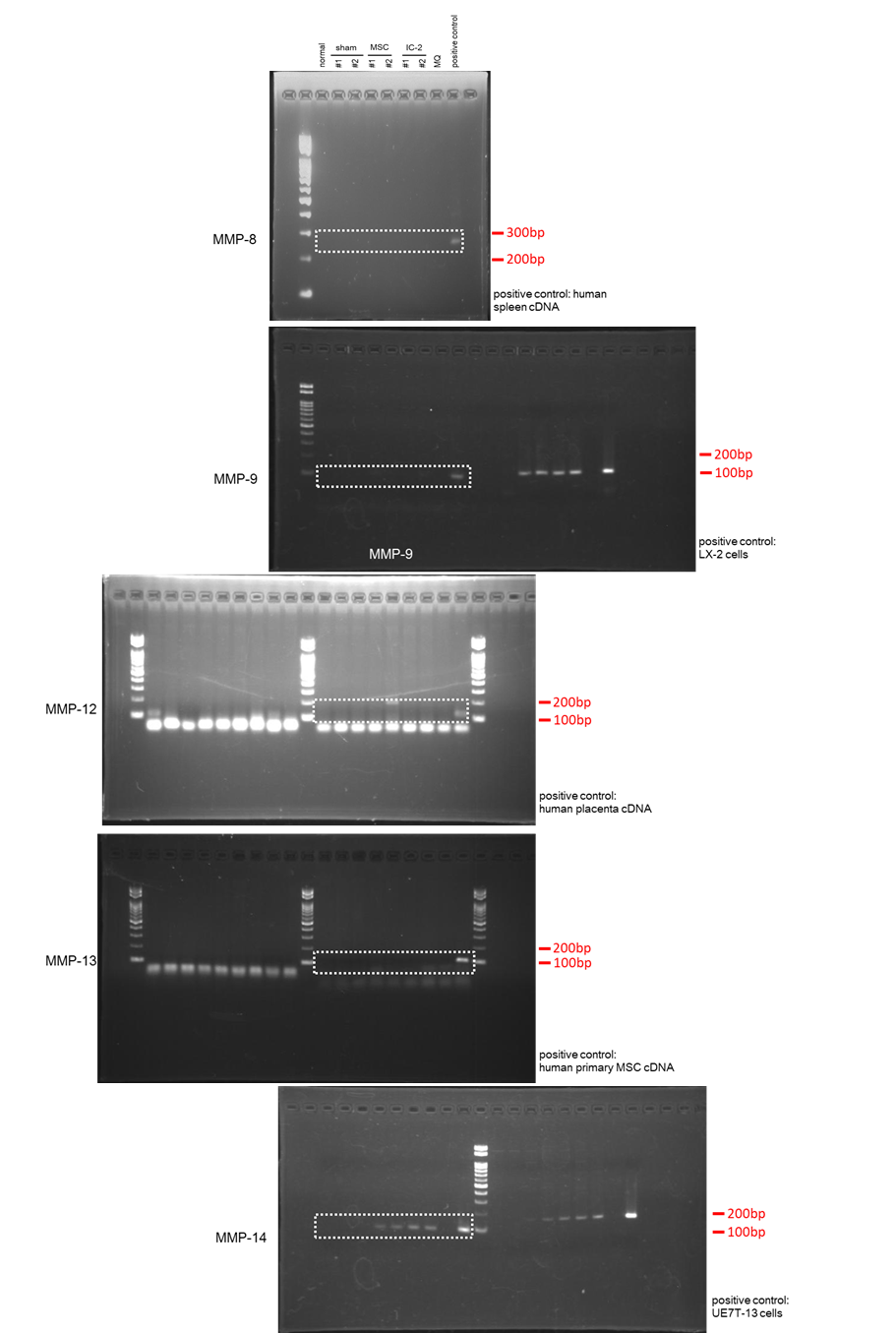
**

**
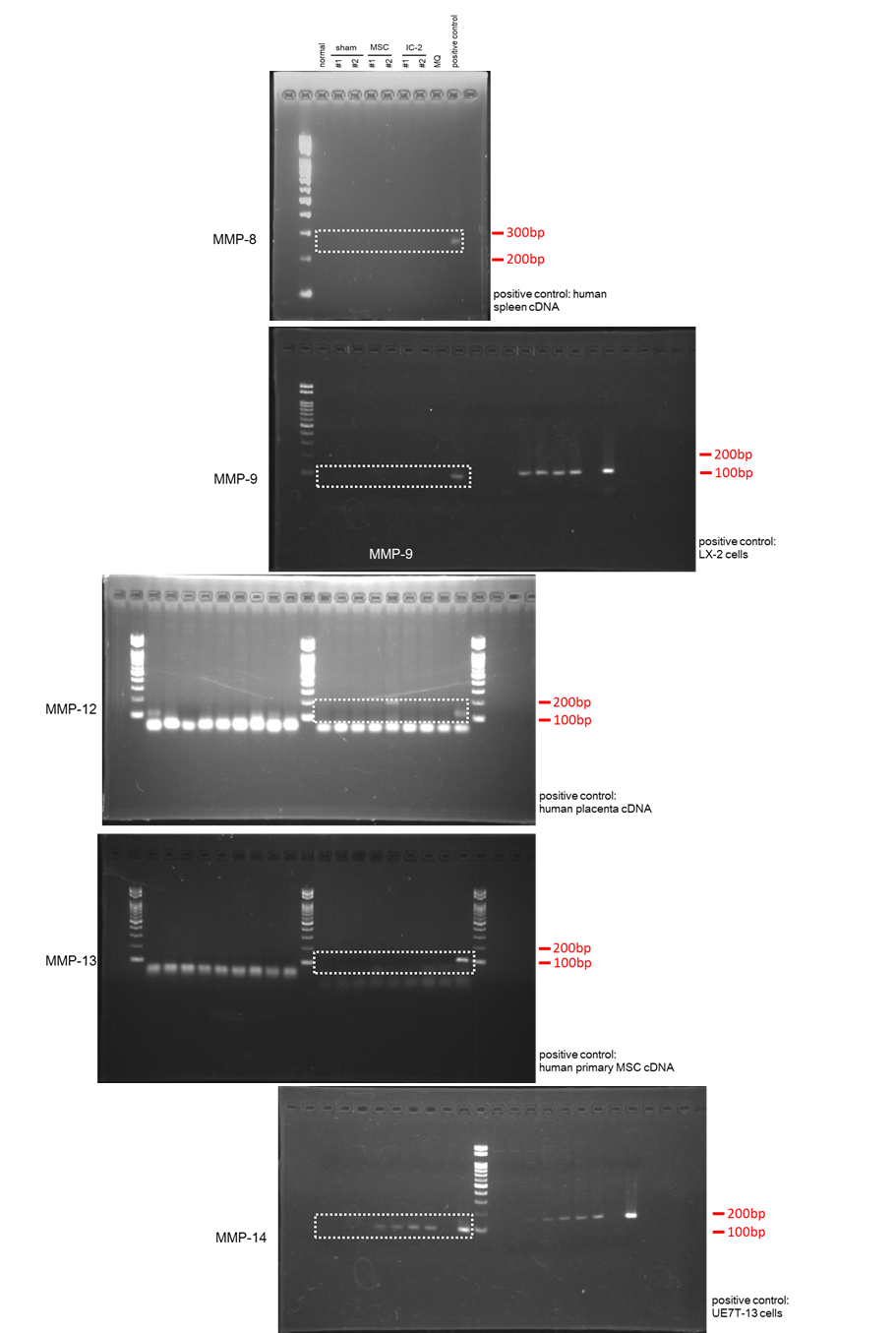

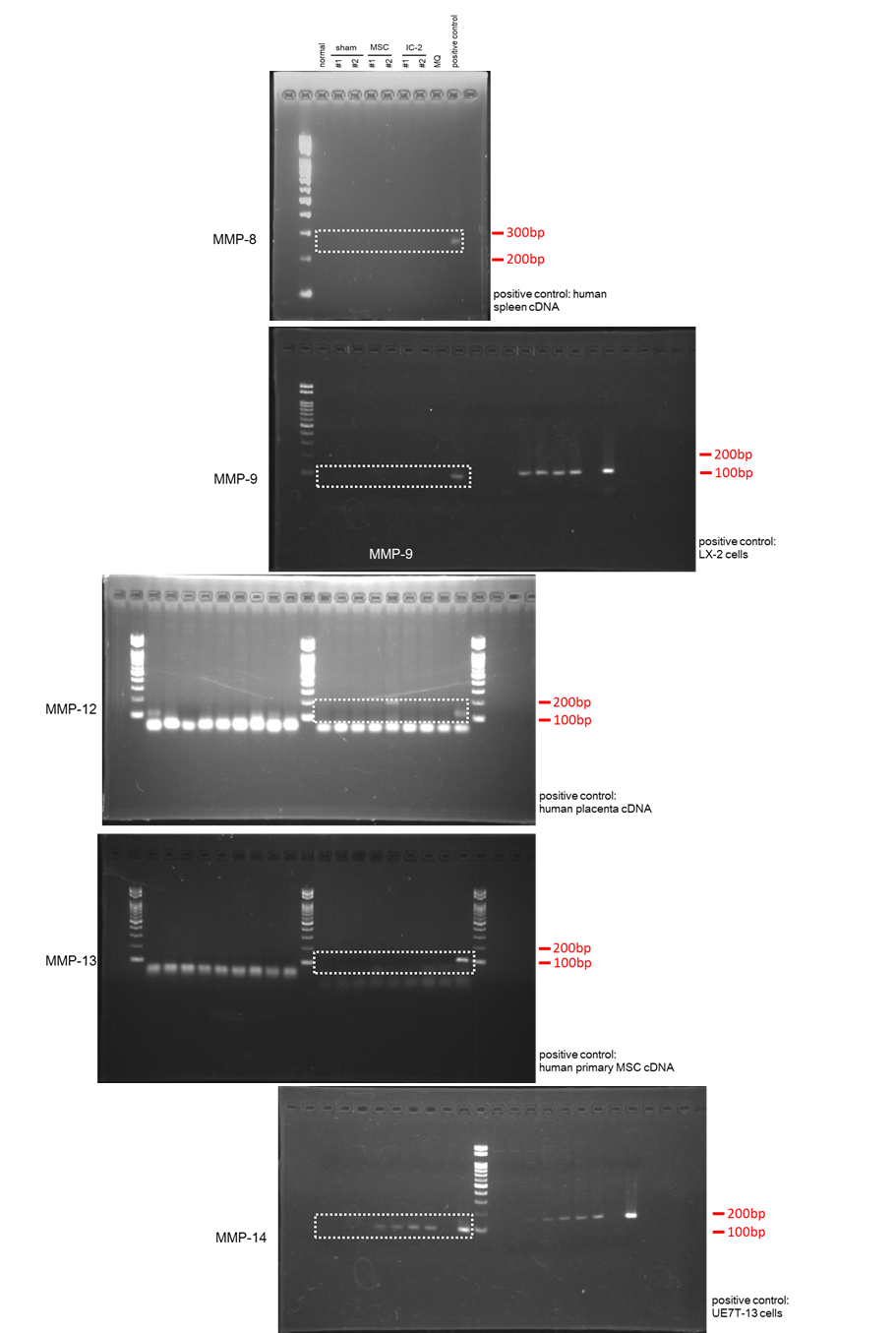
**

**Figure S18. Full length gels of Figure S8.**

White dotted lines show the cropping locations. Brightness and contrast were not changed during processing these gels. Gene Ladder 100 (Wako Pure Chemical Industries Ltd., Osaka, Japan) was applied to marker lanes.

**
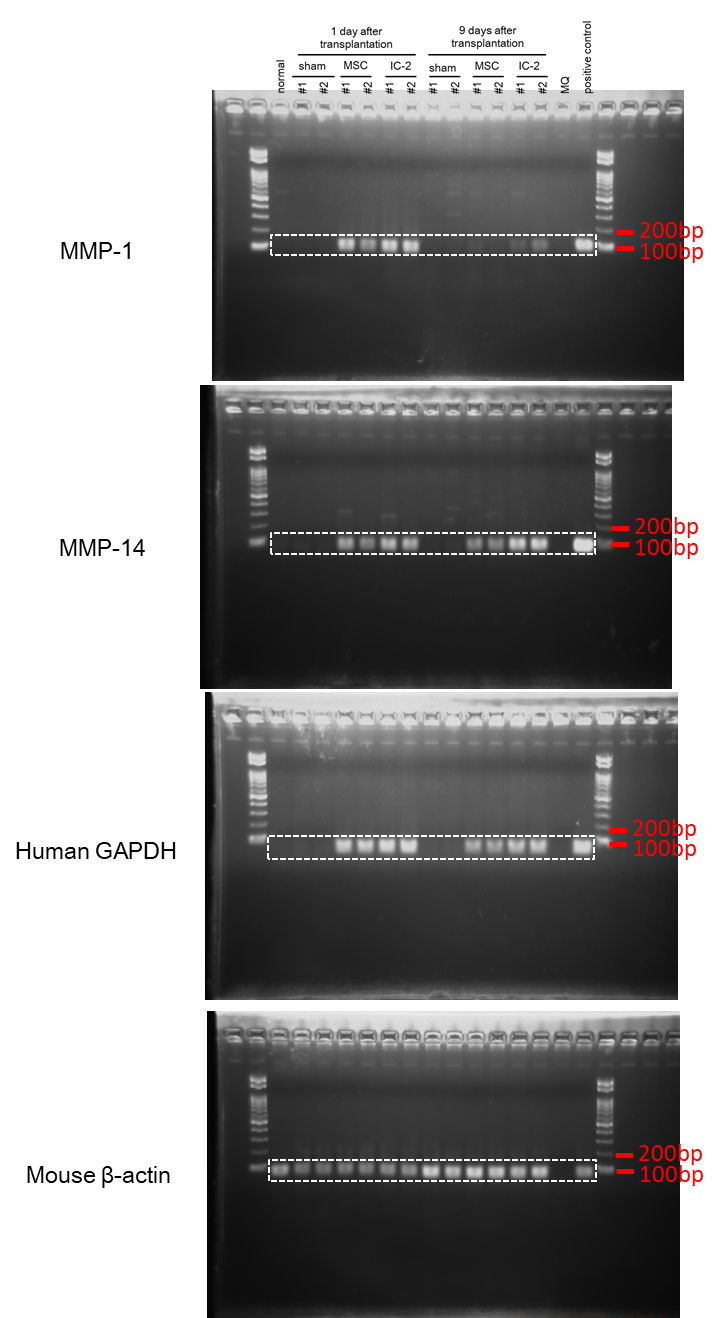
**

**Figure S19. Full length gels of Figure S9A.** White dotted lines show the cropping locations. Brightness and contrast were not changed during processing these gels. Gene Ladder 100 (Wako Pure Chemical Industries Ltd., Osaka, Japan) was applied to marker lanes.

**
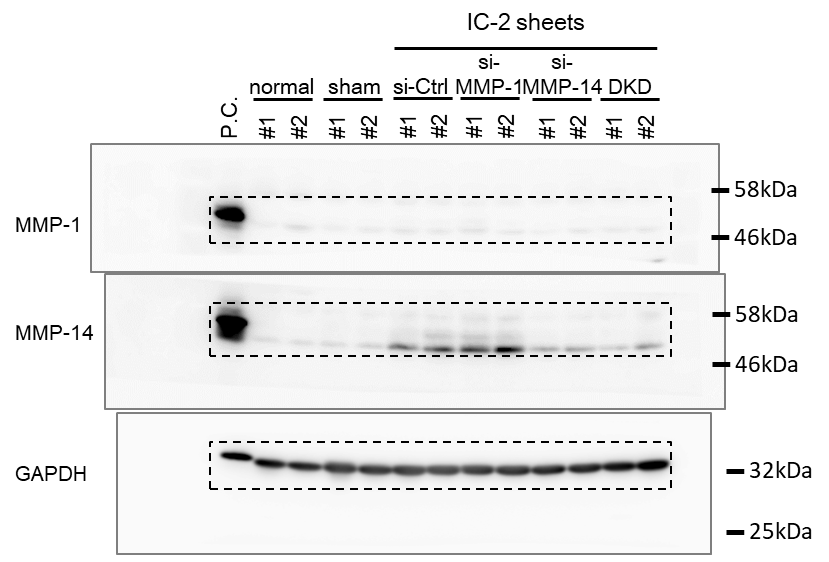
**

**Figure S20. Full length blots of Figure S9B.** Black dotted lines show the cropping locations. Brightness and contrast were not changed during processing these blots.

**Table S1. Antibodies used for immunohistochemistry and Western blot analysis.**

**
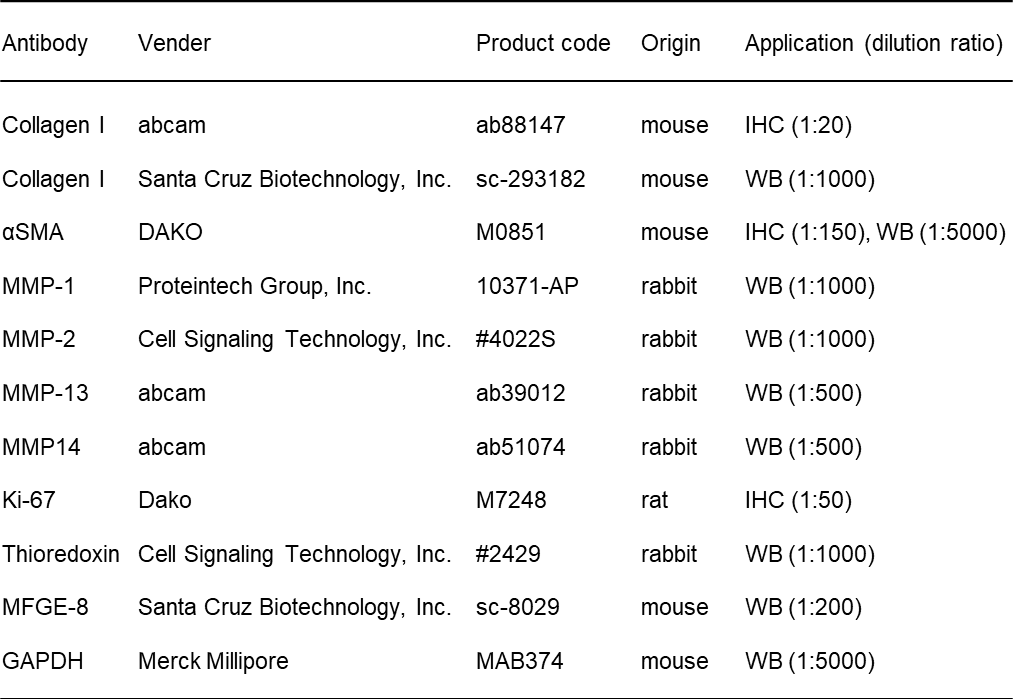
**

IHC, immunohistochemistry; WB, western blotting; αSMA, α smooth muscle actin; MMP, matrix metalloproteinase; GAPDH, Glyceraldehyde 3-phosphate dehydrogenase.

**Table S2. Primers for qRT-PCR and RT-PCR analyses.**


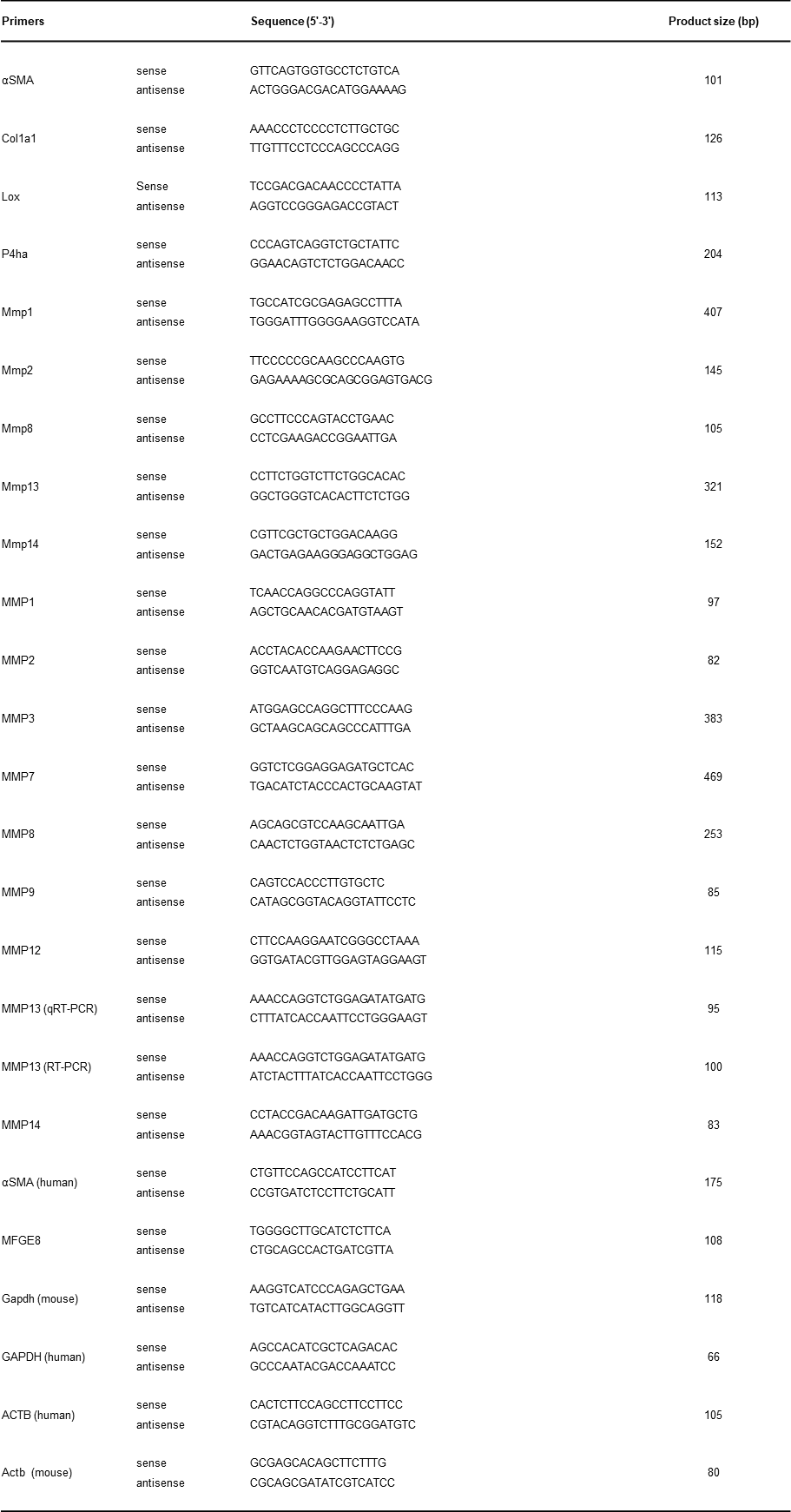


**Supplemental Experimental Procedures**

**Hydroxyproline assay**

We homogenized 100 mg of frozen tissue in 1 mL of deionized water. After three freeze–thaw cycles, homogenates were sonicated at an interval of 15 s for 15 min at 250 W by Bioruptor (Cosmo Bio Co., Ltd., Tokyo, Japan). A small aliquot of homogenate was used to measure protein concentration. Then, 100 µL of the homogenate was added to an equal volume of 12 N HCl and hydrolyzed at 120°C for 16 h. After centrifugation at 800 *g* for 5 min, 10 μL of the supernatant was evaporated to remove HCl. Hydroxyproline content was measured with the hydroxyproline quantification kit (BioVision, CA, USA) according to manufacturer’s instructions.

**Histological analyses**

We prepared 3-μm-thick liver sections and stained them with hematoxylin and eosin. To evaluate fibrosis, Sirius Red staining and azan staining were performed. The 3-μm-thick sections were stained with the picrosirius red staining kit (Polysciences, Inc., Warrington, PA) according to the manufacturer’s protocol. Then, 2-μm-thick sections were stained with azan staining. The ratio of fibrosis area was measured using a BZ-X Analyzer (Keyence Japan, Osaka, Japan) and we measured 10 fields per mouse.

**Immunohistochemistry**

The 3-µm-thick liver sections were deparaffinized and antigens were retrieved by autoclave in a citrate buffer. Endogenous peroxidase activity was blocked by treatment with 3% hydrogen peroxide for 15 min. Primary antibodies and their dilution rate were listed in Supplementary Table 1. The Histofine mouse stain kit and Histofine Simple Stain™ Mouse MAX PO (Rat; NICHIREI BIOSCIENCES INC., Tokyo, Japan) were used for staining with mouse monoclonal primary antibody and primary rat antibody, respectively. Ki-67-positive cell ratios were counted with the inForm Advanced Image Analysis software (PerkinElmer Inc., Waltham, MA).

**Western blot analyses**

Frozen liver tissues, cells, and secretomes were lysed with modified RIPA buffer (50 mM Tris-HCl (pH 7.4), 150 mM NaCl, 0.25% sodium deoxycholate, 0.1% SDS, 1% NP-40, 1 mM EDTA, 5% glycerol, and a protease inhibitor (Complete, Roche Diagnostics GmbH., Mannheim, Germany)). After protein extraction, SDS sample buffer was added and incubated at 37°C for 2 h to analyze collagen expression. Except for those used to detect collagen, proteins dissolved in the SDS sample buffer were incubated at 95°C for 5 min. We applied gel electrophoresis to 3–30 μg of proteins by 8%, 10%, or 15% SDS polyacrylamide gel followed by western blot. Antibodies and their dilution rate are listed in Supplementary Table 1. Chemiluminescent images were acquired by ImageQuant LAS-4000 (GE Healthcare UK Ltd, Little Chalfont, UK) with ECL Prime Western Blotting Detection Reagent (GE Healthcare).

**ELISA analysis**

Frozen liver tissues were lysed with modified RIPA buffer. After protein extraction, lysate was diluted with PBS at 1/100 ratio and used for ELISA analysis to measure type III collagen contents. ELISA Kit for collagen type III alpha 1 (Cloud-Clone Corp., Katy, TX) was used according to the manufacture’s instructions. Measuring protein concentration was used Protein assay dye reagenet (Bio-Rad Laboratories, Hercules, CA) based on the Bradford dye-binding method. Collagen content was normalized with protein concentration.

**RNA extraction, RT-PCR, and qRT-PCR analyses**

Total RNA from the liver tissues or cells was extracted with the TRIzol reagent (Life Technologies Corp.) and subjected to reverse transcription using Superscript II (Life Technologies Corp.) with oligo(dT)_18_ primers. RT-PCR was performed using gene-specific primers and rTaq DNA polymerase (TOYOBO CO., Ltd. Osaka, Japan). qRT-PCR was performed using LightCycler® FastStart DNA Master SYBR Green I (Roche Diagnostics GmbH., Mannheim, Germany) using the LightCycler system (Roche Diagnostics GmbH.). Primers used in RT-PCR and qRT-PCR analyses are listed in Supplementary Table 2.

**MMP assay**

MMP activity was assessed using SensoLyte® 520 MMP1 Assay Kit *Fluorimetric* and SensoLyte® 520 MMP14 Assay Kit (AnaSpec, Inc., CA) according to manufacturer’s instructions.

**Secretome preparation, experiments, and ELISA analysis**

UE7T-13 cells were seeded at a density of 9 × 10^3^ cells/cm^2^ and treated with 15 µM IC-2 on days 1 and 4 after plating. After 1 week of UE7T-13 cell treatment with IC-2, secretomes were prepared. First, the culture media were replaced. The conditioned media were collected 48 h after media replacement and were concentrated using Amicon® Ultra-15 Centrifugal Filters Ultracel-3K (Amicon Ultra; Millipore, Billerica, MA) according to manufacturer’s instructions. Secretomes were examined with western blot analysis. LX-2 HSCs were treated with secretomes at 5% concentration in DMEM/0.1% FBS including 1 ng/mL of recombinant human TGF-β (R&D Systems, Inc., MN). LX-2 cells were harvested and total RNA was extracted 48 h after treatment.

Condition media to prepare secretomes were supplied by Human TXN/ Thioredoxin/ TRX ELISA Kit (LifeSpan BioSciences, Inc., WA).

**Measurement of IC-2 content in the IC-2-treated MSCs.**

UE7T-13 cells were seeded at a density of 9 × 10^3^ cells/cm^2^ onto φ 60mm temperature-responsive polymer-coated culture dishes (CellSeed Inc., Tokyo, Japan) and treated with 15 µM IC-2 on days 1 and 4 after plating. After 1, 3, and 5 days, cell pellets were collected into 1.5 mL tube and frozen at -80℃. 7 days after treatment, cell sheets were detached from dishes by incubating 20℃ for ~30 min and were collected into 1.5 mL tube and frozen at -80℃. IC-2 contents included in these samples were measured at Okayama university. Each samples were added 400 μL of acetonitrile, then the suspension was ultrasonicaed, and centrifuged (10,000 × g, 4 ° C., 10 min) with a tube equipped with millipore filter (15,000 x g, 4 ° C, 45 min). From the supernatant, 200 µL was taken into a new 1.5 mL tube and concentrated under reduced pressure. To this sample was added 100 μL of MeOH and the solution was vortexed and dispensed into a sample tube for LCMS.

The LC-MS system used was the API4000 LC-MS/MS system (Applied Biosystems, Toronto, ON, Canada) consisting of a LC-20AD pump, SPD-20AV UV-Vis spectrophotometric detector, and CTO-20AC column oven (SHIMADZU, Kyoto, Japan). A TSKgel ODS-100V column (2.0 i.d. × 50 mm, 3 µm, TOSOH, Tokyo, Japan) was used at 40 °C. The mobile phase was Acetonitrile/H2O = 50/50 containing 0.1% formic acid, v/v. The flow rate was 0.2 mL/min. th retention time of IC-2 was 5.4–5.7 min.

LX-2 HSCs were treated with 2.3 µM IC-2 in DMEM/0.1% FBS including 2.5 ng/mL of recombinant human TGF-β (R&D Systems, Inc.). Cells were harvested and total RNA was extracted using RNeasy Plus Mini Kit (QIAGEN N.V., Hilden, Germany) 48 h after treatment.

**Statistical analyses**

All values in the present study were expressed as mean ± standard error. Significant differences between groups were analyzed with one-way analysis of variance (ANOVA) post hoc tests (Games–Howell) in SPSS (SPSS Inc., Chicago, IL) unless otherwise noted in legends. A p-value of <0.05 was considered to be significant.
